# Chemoinformatics-based enumeration of chemical libraries: a tutorial

**DOI:** 10.1186/s13321-020-00466-z

**Published:** 2020-10-27

**Authors:** Fernanda I. Saldívar-González, C. Sebastian Huerta-García, José L. Medina-Franco

**Affiliations:** 1 DIFACQUIM Research Group, School of Chemistry, Department of Pharmacy, Universidad Nacional Autónoma de México, Avenida Universidad 3000, 04510 Mexico, Mexico; 2 School of Chemistry, Department of Pharmacy, Universidad Nacional Autónoma de México, Avenida Universidad 3000, 04510 Mexico, Mexico

**Keywords:** Chemical enumeration, Chemoinformatics, Combinatorial libraries, DOS synthesis, Drug design, Education, KNIME, Python

## Abstract

Virtual compound libraries are increasingly being used in computer-assisted drug discovery applications and have led to numerous successful cases. This paper aims to examine the fundamental concepts of library design and describe how to enumerate virtual libraries using open source tools. To exemplify the enumeration of chemical libraries, we emphasize the use of pre-validated or reported reactions and accessible chemical reagents. This tutorial shows a step-by-step procedure for anyone interested in designing and building chemical libraries with or without chemoinformatics experience. The aim is to explore various methodologies proposed by synthetic organic chemists and explore affordable chemical space using open-access chemoinformatics tools. As part of the tutorial, we discuss three examples of design: a Diversity-Oriented-Synthesis library based on lactams, a bis-heterocyclic combinatorial library, and a set of target-oriented molecules: isoindolinone based compounds as potential acetylcholinesterase inhibitors. This manuscript also seeks to contribute to the critical task of teaching and learning chemoinformatics.

## Introduction

Hit identification is the starting point and one of the most crucial stages of small-molecule drug discovery [1]. One approach to increase the likelihood of finding new hit compounds is presented by the computational generation of virtual chemical libraries to be used in various virtual screening methods. Thus, many researchers are developing new de novo chemical libraries and libraries “make-on-demand” by different in silico approaches [2]. For example, GDB‐17 generated by Reymond et al. is a chemical library that explores the chemical space broadly by enumerating more than 160 billion organic small molecules with up to 17 atoms [3]. Another example is the 95 million compounds in the virtual library CHIPMUNK (CHemically feasible In silico Public Molecular UNiverse Knowledge base) that were enumerated by performing a selected set of reactions widely used in traditional combinatorial chemistry [4]. Other examples of virtual libraries based on prevalidated or reported reactions, as well as accessible chemical reagents developed by pharmaceutical companies are BI-Claim developed by Boehringer Ingelheim [5], Eli Lilly’s Proximal Collection [6], Pfizer global virtual library (PGVL) [7], and Merck’s Accessible inventory (MASSIV) [8]. This approach was also used by chemical vendors to generate “make-on-demand” virtual libraries such as the “Readily Accessible” (REAL) Database and REAL Space being the largest synthetic accessibility-based virtual compound collections to date [9].

In general, virtual libraries address the need to improve the quality of compounds to identify efficiently lead compounds [10]. In this context, the size, the structural complexity, and the diversity of the virtual libraries play a key role in increasing the chance of a successful drug discovery and development outcome [11]. Another critical aspect of virtual libraries’ generation is that the compounds obtained must have some novelty, and most importantly, they must be synthetically feasible. This strategy is particularly attractive to build libraries for difficult and emerging molecular targets [12].

The construction of a virtual chemical compound can be done in a variety of ways. For example, using a known reaction schema and available reagents, based on functional groups, by de novo*-*based design, by morphing/transformation, or by decorating a molecular graph [13].

Different tools have been developed to enumerate virtual libraries and are summarized in Table 1. Some of these tools replace a predetermined central unit of a molecule, such as Molecular Operating Environment (MOE) [14] and Schrödinger [15]. Other approaches are based on combinatorial enumeration from specifications of central scaffolds with connection points and lists of R groups such as SMILES or standard data files (SDF) like Library synthesizer [16] or Nova [17]. Few tools allow the user to enter a list of pre-validated reactions to generate virtual libraries like Reactor [18], DataWarrior [19], and KNIME [20]. These tools have the advantage of being freely accessible. For Reactor, an academic license can be requested. Our research group recently developed D-Peptide Builder, a free webserver to enumerate combinatorial peptide libraries. The user can build linear or cyclic peptide libraries with *N*-methylated or non-methylated amino acids [21, 22].

**Table 1:** Examples of chemoinformatic tools available to enumerate virtual chemical libraries

Tool	Main features	References
Free tools		
RDKit	Library enumeration is based on generic reactions and that for every one of its generic reactants a list of real reactant structures is provided	[23]
DataWarrior	Enumerated product structures are generated from a given generic reaction and that for every one of its generic reactants a list of real reactant structures is provided	[19]
KNIME	Library enumeration is based on generic reactions, where a list of reagent structures is provided for each of its generic reagents	[24]
Library synthesizer	Enumerated chemical libraries from specifications of central scaffolds with connection points and lists of R groups	[16]
D-Peptide Builder	A chemoinformatic tool to enumerate combinatorial libraries of up to pentapeptides, linear or cyclic, using the natural pool of 20 amino acids. The user can use non‐ and/or N‐methylated amino acids. The server also enables the rapid visualization of the chemical space of the newly enumerated peptides in comparison with other libraries relevant to drug discovery and preloaded in the server	[21]
SmiLib v2.0	Tool for rapid combinatorial library enumeration in the flexible and portable SMILES notation. Combinatorial building blocks are attached to scaffolds by means of linkers, this allows for the creation of customized libraries using linkers of different sizes and chemical nature	[25]
GLARE (Global Library Assessment of REagents)	Allows to optimize reagent lists for the design of combinatorial libraries	[26]
Comercial tools		
Reactor (ChemAxon)	Library enumeration is based on generic reactions combined with reaction rules; therefore, it is capable of generating chemically feasible products without preselection of reagents	[18]
Molecular Operating Environment (MOE)	Scaffold Replacement: New chemical compounds are generated by replacing a portion of a known compound (the scaffold), while preserving the remaining chemical groups QuaSAR_CombiGen: A single combinatorial product is constructed by attaching R-groups to a scaffold at marked attachment points, called ports. The entire combinatorial library is enumerated by exhaustively cycling through all combinations of R-groups at every attachment point on every scaffold	[14]
Schrödinger	Core hopping: Create libraries by substituting one or several attachments on a core structure with fragments from reagent compounds	[15]
Nova (Optibrium)	Enumerated chemical libraries from specifications of central scaffolds with connection points and lists of R groups	[17]

The pre-validated reactions strategy will result useful for synthetic organic chemists, aimed to explore all possible compounds obtained through the reactions or design approaches developed within their research groups or reported in the literature. However, several experimental research groups do not have access to commercial software and/or do not have a background in informatics to rapidly use the open-source tools to enumerate chemical libraries.

This manuscript aims to present and discuss a step-by-step tutorial to enumerate chemical libraries using open-access chemoinformatics tools. As part of the tutorial, three chemical libraries’ design approaches were developed. One using the DOS Build/Couple/Pair approach, the second exemplifies the design of a bis-heterocyclic combinatorial library. The third is the design of isoindolinone-based compounds as putative acetylcholinesterase (AChE) inhibitors. The design and construction of these libraries are explained step by step. This manuscript also aims to contribute to the critical task of learning chemoinformatics [27].

## Chemical data formats

### Single chemical structures

**Fig. 1:**
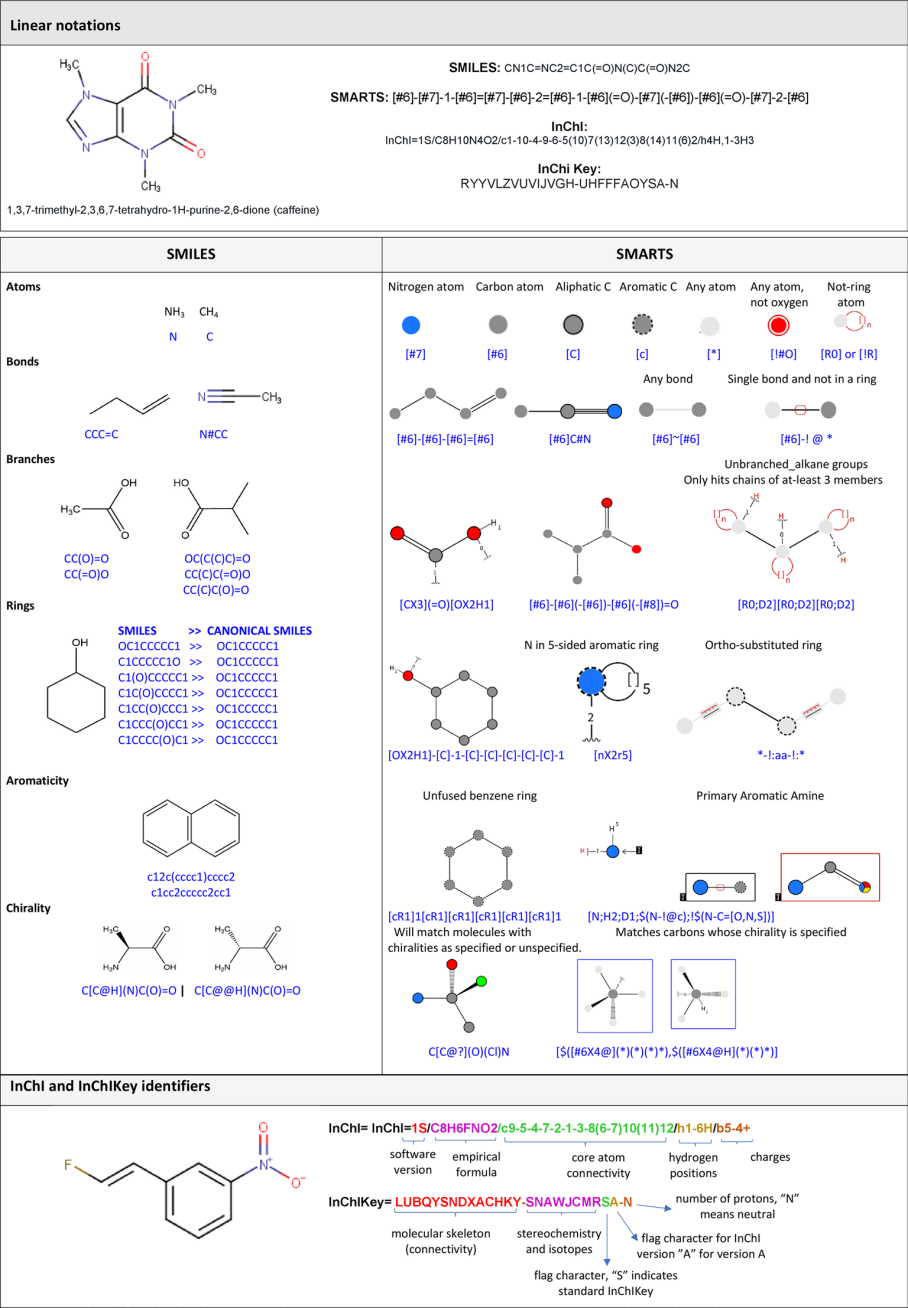
SMILES, SMARTS, InChI and InChIKey concepts. Examples for the illustration of basic SMILES, SMARTS, InChI, and InChIKey syntax rules are provided. SMARTS representations were made in SMARTviewer [35]. InChI and InChIKey identifiers are displayed for caffeine and 1-[(*E*)-2-fluorovinyl]-3-nitrobenzene

As in almost every task in chemoinformatics, molecular representation is a key aspect to consider during the enumeration of chemical compounds [28]. Probably the most well-known description of compounds is the two-dimensional (2D) graphical representation. There are currently many programs to help draw chemical structures and facilitate the storage and interconversion between standard file formats. Some of these software programs have free academic versions such as MarvinSketch [29] and ACD/ChemSketch [30], and others are commercial such as ChemDraw [31], Schrödinger [15], and MOE [14], to name a few [32]. Three-dimensional (3D) structures are also widely used, especially now that numerous computer programs have been developed to calculate and visualize them. These representations provide a powerful and intuitive tool for understanding many aspects of chemistry. However, they have limitations, especially when it comes to everyday tasks in chemoinformatics that require storage and handling a vast number of compounds [33]. In these applications, molecular information is typically represented by the linear notation [34]. Hereunder, we describe some of the most commonly used linear notations to enumerate chemical structures: SMILES, SMARTS, InChi, and InChikeys. Intuitive examples illustrating the general concepts of such linear notations are shown in Fig. 1.

### SMILES

Short and readable descriptions of molecular graphs are linear notations. A clear example is the broadly used Simplified Molecular Input Line System (SMILES), which captures a molecules’ structure in the form of an unambiguous text string using alphanumeric characters. They allow the efficient storage and fast processing of large numbers of molecules. The SMILES notation uses the following basic rules for encoding molecules [36, 37]:

1. Atoms are represented by their atomic symbols. Hydrogen atoms saturating free valences are not represented explicitly.
2. Neighboring atoms stand next to each other, and bonds are characterized as being single (-), double ( =), triple (#), or aromatic (:). Single and aromatic bonds are usually omitted.
3. Enclosures in parentheses specify branches in the molecular structure.
4. For the linear representation of cyclic structures, a bond is broken in each ring and the connecting ring atoms are followed by the same digit in the textual representation.
5. Atoms in aromatic rings are indicated by lower case letters. In some cases, there may be problems with aromaticity perception.

Although SMILES strings are unambiguous in describing chemical structures, they are not unique because multiple valid SMILES representations exist for the same molecular graph. Canonical SMILES strings are often used to ensure the uniqueness of molecules in a database. In principle, canonical SMILES strings can be used to identify duplicated compounds, but in practice, canonicalization differs between programs. For more consistent, documented, and standardized duplicated removal, the IUPAC International Chemical Identifier (InChi, InChiKey) [38] is recommended. Another aspect that must be taken into account when using SMILES is the handling of tautomers. Tautomerization can lead to alternative SMILES strings for the same ligand, and inconsistencies SMILES interpretation can lead to inconsistencies in tautomer representation. Several programs can enumerate canonical tautomers (e.g., Accelerys, OpenEye, and Schrödinger), and this is recommended for the consistent processing of molecules.

### SMARTS

SMILES Arbitrary Target Specification (SMARTS) is a language developed to specify substructural patterns used to match molecules and reactions. Substructure specification is achieved using rules that are extensions of SMILES. In particular, the atom and bond labels are extended to also include logical operators and other special symbols, which allow SMARTS atoms and bonds to be more inclusive [39]. This notation is especially useful for finding molecules with a particular substructure in a database. SMARTS can also be used to filter out molecules with substructures that are associated with toxicological problems [40] or that appear as frequent hitters (promiscuous compounds) in many biochemical high-throughput screens (Pan Assay Interference Compounds, PAINS) [41]. Other applications are the separation of active from inactive compounds and the evaluation of ligand selectivity. The characterization of chemical reaction centers has been described by Rarey et al. [42], through the development of a new algorithm called SMARTSminer, which allows the automatic derivation of discriminative SMARTS patterns from sets of pre-classified molecules.

The SMARTS language provides several primitive symbols describing atomic and bond properties beyond those used in SMILES (atomic symbol, charge, and isotopic specifications). Table 2 lists the atomic and bond primitives used in SMARTS [39].

**Table 2:** SMARTS atomic and bond primitives

SMARTS atomic primitives	SMARTS bond primitives
*: any atom a: aromatic A : aliphatic D<n>: degree, <n> explicit connections H<n>: total-H-count, <n> attached hydrogens h<n>: implicit-H-count, <n> implicit hydrogens R<n>: ring membership, in <n> SSSR rings r<n> ring size, in smallest SSSR ring of size <n> v<n>: valence, total bond order <n> X<n>: connectivity, <n> total connections x<n>: ring connectivity, <n> total ring connections +<n>: positive charge, +<n> formal charge -<n>: negative charge, +<n> formal charge #n : atomic number @: chirality	-: single bond (aliphatic) /: directional bond "up" \: directional bond "down" /?: directional bond "up or unspecified" \?: directional bond "down or unspecified" = : double bond #: triple bond : : aromatic bond ~: any bond (wildcard) @ : any ring bond

Atom and bond primitive specifications may be combined to form expressions by using logical operators. SMARTS examples can be found on Daylight's web site [43].

Because chemical pattern representations are relatively new, the number of interfaces where the user can graphically create patterns is limited. Examples of editors to handle SMARTS notation are MarvinSketch [29], JSME [44], SMARTeditor [45], and the PubChem’s Sketcher web editor [46, 47]. A comparison between these editors was described by Schomburg et al. [45].

### InChI and InChI Keys

InChI is the International Chemical Identifier developed under IUPAC’s auspices, the International Union of Pure and Applied Chemistry, with principal contributions from NIST (the U.S. National Institute of Standards and Technology) and the InChI Trust [38]. The InChI objective is to establish a unique label for each compound and allow an easier linking of diverse data compilations. This notation resolves many of the chemical ambiguities not addressed by SMILES, particularly concerning stereocenters, tautomers, and other valence model problems. However, InChIs are difficult to read and interpret by humans in most cases. InChIs comprise different layers and sub‐layers of information separated by slashes (/). Each InChI string starts with the InChI version number, followed by the main layer. This main layer contains sub‐layers for empirical formula, atom connections, and hydrogen atoms positions. The identity of each atom and its covalently bonded partners provide all of the information necessary for the main layer. The main layer may be followed by additional layers, for example, for the charge, isotopic composition, tautomerism, and stereochemistry [35].

The InChIKey is a fixed-length (27-character) condensed digital representation of an InChI, developed to make it easy to perform web searches for chemical structures. The first block of 14 characters for an InChIKey encodes core molecular constitution, as described by a formula, connectivity, hydrogen positions, and charge sublayers of the InChI main layer. The other structural features complementing the core data—namely exact positions of mobile hydrogens, stereochemical, isotopic, and metal ligands, whichever are applicable—are encoded by the second block of InChIKey. The possible protonation or deprotonation of the core molecular entity (described by the protonation sublayer of the InChI main layer) is encoded in the very last InChIKey flag character. Further details of InChIKey are described here https://www.inchi-trust.org.

## Chemical reactions

Representing chemical reactions is much more complicated than representing single structures [48]. To represent chemical reactions is of particular importance to identify the reactants, products, and if it wants to represent reactions more generically, it is required to determine the reaction center, that is, the collection of atoms and bonds that are changed during the reaction [49], so that the substructural transformation can be described by specifying the reactive substructures in the reagent and the product. To this end, Daylight [50] has developed SMILES so that they can be used to describe reactions, SMARTS for reaction queries, and SMIRKS to describe transformations [51]. For its part, IUPAC has also been developing a non-proprietary, international identifier for reactions "RInChI" [52]. The RInChI project’s objective is to create a unique data string record and structure detailed information on reaction processes, using InChI software. These approaches are powerful and flexible, allowing for the inclusion of various information, including atom mapping.

To understand the scope of these approaches and the importance of atom mapping, suppose we look for reactions that let us obtain an alcohol from a carbonyl group, such as an ester. If we look for reactions in which there is a carbonyl group in the starting material and alcohol in the product, this search may produce undesirable results, where there is another carbonyl group or alcohol in the starting material. Still, the reaction does not change (see Table 3, Reaction 1). Atom-to-atom mapping ensures that both the carbonyl and alcohol groups are at the reaction site. However, it is essential to note that atom mapping depends on the reaction mechanism, as shown in reactions 2 and 3 of Table 3.

**Table 3:**
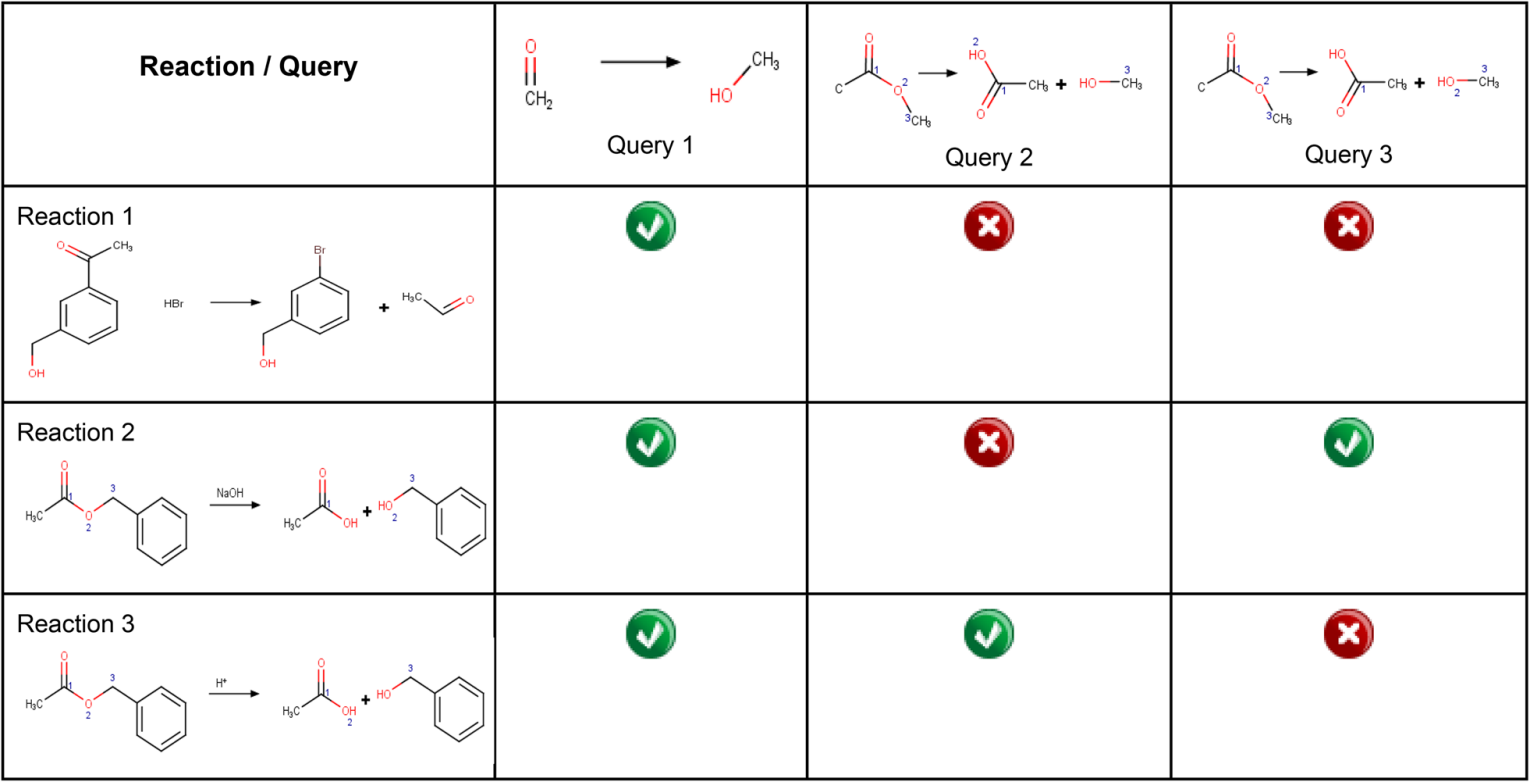
Examples of reaction queries

To accurately capture a generic reaction, there are two requirements. The first is the actual set of changes in the molecule that occurs during the reaction (captured with changes in atoms and bonds). The second is the indirect effects of activating and deactivating groups near the reaction site [39].

Within the Daylight’s system, the indirect effects on a generic reaction are most appropriately expressed with the SMARTS query language. However, SMARTS have been designed for efficient querying of reaction databases, and they do not have the other requirements to accurately capture a generic reaction. SMIRKS accomplishes this by concisely expressing the atom and the list of bond changes of a reaction, as well as the indirect effects of activating and deactivating groups near the reaction site. SMIRKS is a hybrid of SMILES and SMARTS and can be used to represent reaction mechanisms, resonance, and general modifications of molecular graphs [53, 54]. It is a restricted version of reaction SMARTS with a set of rules that act as constraints. A comparison between SMILES, SMARTS, and SMIRKS to represent chemical reactions is described in Table 4.

**Table 4:** Comparison between SMILES, SMARTS and SMIRKS to represent chemical reactions

	SMILES	SMARTS	SMIRKS
Representation	*Reactant > Agent > Product* In some cases the presence of agents can be omitted *Reactant > > Product*	A reaction query may be composed of optional reactant, agent, and product parts, which are separated by the " > " character *Reactant > Agent > Product* *Reactan > > * * > Agent > * * > > Product* *Query*	*Reactant > > Product*
Example	CC(= O)O.OCC > [H +].[Cl-].OCC > CC(= O)OCC	> > [#6][CX3](= O)[#6] This query returns reactions in which the product contains ketones	[C:1]([O,Cl:5]) = [O:2].[N:3][H:4] > > [N:3][C:1] = [O:2].[*:5][H:4][C]([O,Cl]) = [O].[N][H] > > [N][C] = [O].[*][H] The use of the SMARTS [O,Cl] allows oxygen or chlorine
Characteristics	The map is always the last part of the atom expression delimited by a colon and it is optional If hydrogen is mapped, it is also "special" and must be shown (hydrogens are normally omitted from SMILES)	Atom map is optional Any valid Reaction SMILES is a valid SMARTS query Any valid Molecule SMARTS can be a component of a Reaction Recursive SMARTS supports only molecule expressions All valid SMIRKS are valid reaction queries	Atoms can be added or deleted during a transformation Atomic SMARTS expressions can be used for atoms directly involved in the reaction (the reaction center) Stoichiometry is defined to be 1–1 for all atoms in the reactant and product for a transformation Explicit hydrogens that are used on one side of a transformation must appear explicitly on the other side of the transformation must be mapped Bond expressions must be valid SMILES (no bond queries allowed) Atomic expressions may be any valid atomic SMARTS expression for nodes where the bonding (connectivity and bond order) does not change
Use	To represent specific reactions between specific reactants yielding specific products	SMARTS are used for searching reactions	SMIRKS are used to represent generic chemical transformations
Applications	Store a library of reactions of interest (these might be a record of reactions that have been carried out at a company, a set of reaction plans in an academic research group, or even a set of hypothetical reactions that might never succeed in the laboratory)	Retrieve specific searches Avoid uninteresting results Reaction classification and categorization	Using SMIRKS to represent chemical transformations, reaction specifications can be stored in the database Structures can be transformed and combined (reacted) to produce new structures

### Chemical reaction database systems

Reaction databases store information that can help create a data-rich environment in the early stage of pharmaceutical process–product development. With this information, various improvements to the initial selection process can be established, which can be seen mainly reflected in a decrease in cost and time required. For example, it can compare different reactions to produce the same product, analyze different ways to carry out a specific transformation of a functional group, and specify reaction's conditions. It can also evaluate the reaction path in terms of performance, cost, and sustainability [55].

Searching for reactions and retrieving relevant information from a chemical reaction is a complex task and involves searching for chemical structures of reagents or products (complete or partial), transformation information (reaction centers), description of reactions (the type of reaction, general comments), and numerical data about the experimental reaction (yield, selectivity, reaction conditions, etc.). For this reason, efforts have been made to classify databases concerning their search reaction information. The criteria that have been established are the following [56].

i) Each reaction is an individual record in the database (detailed and graphical). The reaction must be retrieved from the database as a detailed record (reagents, products, stoichiometry, etc.). It can also be extracted as a graphical representation where the reaction scheme is shown. In many databases, the reaction is represented in a graphical form.
ii) Structural information for target product as well as substrates.
iii) Reaction centers are reliably assigned and searchable. The reaction center of a reaction is the collection of atoms and bonds changed during the reaction [49].
iv) Reaction components must be searchable. Information for the components involved in the reaction such as reagent, catalysts, solvents, etc.
v) Multistep reactions. In the case of multistep reactions, all reactions (individual and whole pathway) must be searchable.
vi) Reaction conditions. Conditions such as pH, temperature, pressure, etc. should be searchable by exact and suitable values.
vii) Reaction classification. The type of reaction (i.e., esterification) should be searchable.
viii) Post-processing of the database contents. Export of the retrieved reaction data in other tools (i.e., MS Excel).

The main reaction databases that help organize, store, and retrieve data have been described by Papadakis et al. [55]. The CASREACT reaction database [57, 58] stands out as containing the most significant number of reported reactions, approximately 123 million single-step and multi-step reactions, dating from 1840 to the present. This database can be used to provide information on different ways to produce the same product (single-step or multi-step reactions), used for applications of a particular catalyst, and various ways to carry out specific functional group transformations [59]. Another reaction database is REAXYS [60], based on Elsevier’s industry-leading chemistry databases that include data for more than 49 million reactions, dating from 1771 to the present. It includes many compounds (organic, inorganic, and organometallic) and experimental reaction details (yield, solvents, etc.). It is searchable for reactions, substances, formulas, and data such as physicochemical properties data, spectra. Additionally, the REAXYS database can be used for synthesis route planning [61].

WebReactions from Open Molecules [62] is a good example of an open access reaction database. It introduces a new concept for retrieving reactions from a large database in which reactions are indexed by the bond changes that occur and the effect of the surrounding groups on such bonds in aspects like rate, hindrance, or resistance to change. Unlike conventional reaction databases working on reaction substructure search, WebReactions rather perform a customizable reaction similarity search focusing on the reaction center.

**Fig. 2:**
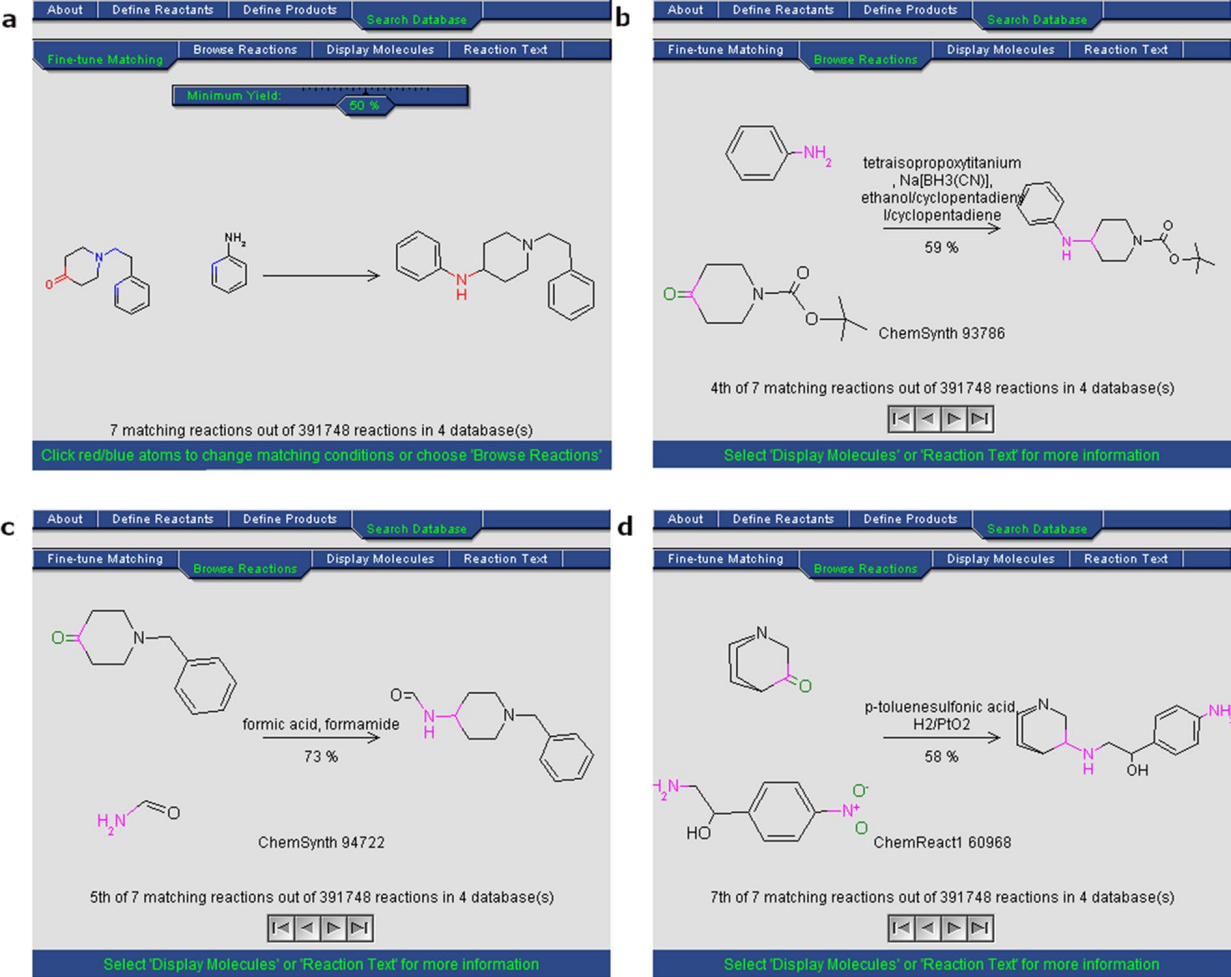
Searching the reductive amination involved in the synthesis of fentanyl in WebReactions. **a** Reaction input and fine-tuning. **b**–**d** Example results

The database entries are taxonomically indexed with these successively nested subheadings: a rigorous digital generalization of the reaction class and type, the nature of substitution surrounding the reaction center, the nature of entering and/or leaving groups, features in the reactant which remains unchanged in the reaction. For example, the synthesis of fentanyl, a potent opioid analgesic [63], and its synthetic derivatives involve a reductive amination that can be searched for in WebReactions [64]. As shown in Fig. 2a, once the reaction of interest is drawn, reaction centers are defined (red), and a minimum yield and characteristics of surrounding atoms can be established. In this case, there are seven matching reactions, three examples are in Fig. 2b–d, which show how similar reactions could be carried out under different reducing agents and conditions. Each result provides the reactant, product, and catalyst, and the original paper's reference. A synthetic laboratory may select candidate reactions based on the highest possible yield, or what resources (such as reagents) are readily available.

## Freely available and open-source tools for the computational-aided design of chemical libraries

The virtual enumeration of chemical reactions is a powerful tool in systematic compound library design. The exploration of virtual chemistry is bounded only by the human imagination and the capabilities of computers. By using reactions deposited in chemical reaction databases, a large number of virtually obtained compounds can be accessed. Therefore, careful planning of these reactions is of utmost importance to influence the products obtained in these experiments. Until now, computer-based methods have considered generating compounds to address issues such as the diversity of chemical libraries [8, 65], the design of drug-like or focused libraries [66], and on making and identifying compounds for high-throughput screening strategies [67].

For the efficient design of chemical libraries, it is important to keep in mind the type of compounds to obtain to later evaluate the strategic bonds and select a strategy to use. The choice of strategy to use will largely depend on the ease with which this strategy has to be adopted by medicinal chemists and the additional problems to be covered (structural features, physicochemical properties, and diversity). The synthesis strategy that has been mostly addressed to generate virtual libraries is combinatorial chemistry, however, other approaches such as diversity-, biology-, lead-, or fragment oriented synthesis can be easily implemented [68]. In this part, it is essential to focus on well-characterized reactions, to avoid the bottleneck in current computational approaches to drug design: the assessment of synthetic accessibility [69].

Another pragmatic way to improve compound quality while enhancing and accelerating drug discovery projects is to access and propose a high quality, novel, diverse building block collection [70]. Guidelines have been developed that provide more specific guidance to medicinal chemists and help prioritize the synthesis of compounds. Among these guidelines is the proposed 'rule of 3′ (MW ≤ 300; logP -3 to 3; HBA ≤ 3; HBD ≤ 3; tPSA ≤ 60, Rotatable bonds ≤ 3) to guide fragment selection for fragment-based lead generation [71] and the 'rule of 2′ (MW < 200, clogP < 2, HBD 2, HBA 4) to design novel reagents for drug discovery projects [70]. These guidelines can help not only prioritize reagents but also target libraries to compounds with optimal physicochemical properties for drug design. Databases such as ZINC DB [72], Asinex [73], Life Chemicals [74], and Maybridge [75] can be used to access and download catalogs of commercially available starting materials.

In order to exemplify the points above, this section focuses on creating libraries of chemical compounds from public data sources, generated using different synthetic strategies and various open-access tools like RDKit, KNIME, and DataWarrior. The designed libraries are synthetically accessible as the design approach was based on feasible reactions and existing reagents. However, this does not mean that the obtained compounds are easy or cheap to carry out. If an approach based on known reaction schemes was not applied, it would be necessary to evaluate the synthetic feasibility of the possible synthetic routes or the products’ accessibility, which we discuss further in this manuscript.

## Design of a library of bis-heterocycles obtained with click chemistry using Python and the RDKit package

As medicinal chemists try to mimic the core elements of a wide range of natural products such as nucleic acids, amino acids, carbohydrates, vitamins, and alkaloids, heterocycles have become a standard structural unit in drug discovery. These structures allow modulating important drug properties such as potency and selectivity through bioisosteric replacements, lipophilicity, polarity, and aqueous solubility [76].

Click chemistry provides a means for the rapid exploration of the chemical universe enabling rapid structure–activity relationships (SAR) profiling through the generation of analog libraries. Click chemistry is wide-ranging, owing to strongly driven, highly selective reactions of broad scope, allowing a much greater diversity of block structures to be used [77]. Huisgen’s copper-(I) catalyzed 1,3-dipolar cycloaddition of alkynes and azides yielding triazoles is the premier example of a click reaction [78], due to the accessibility of azides and alkynes, highly diverse, unambiguous libraries become available quickly.

**Fig. 3:**
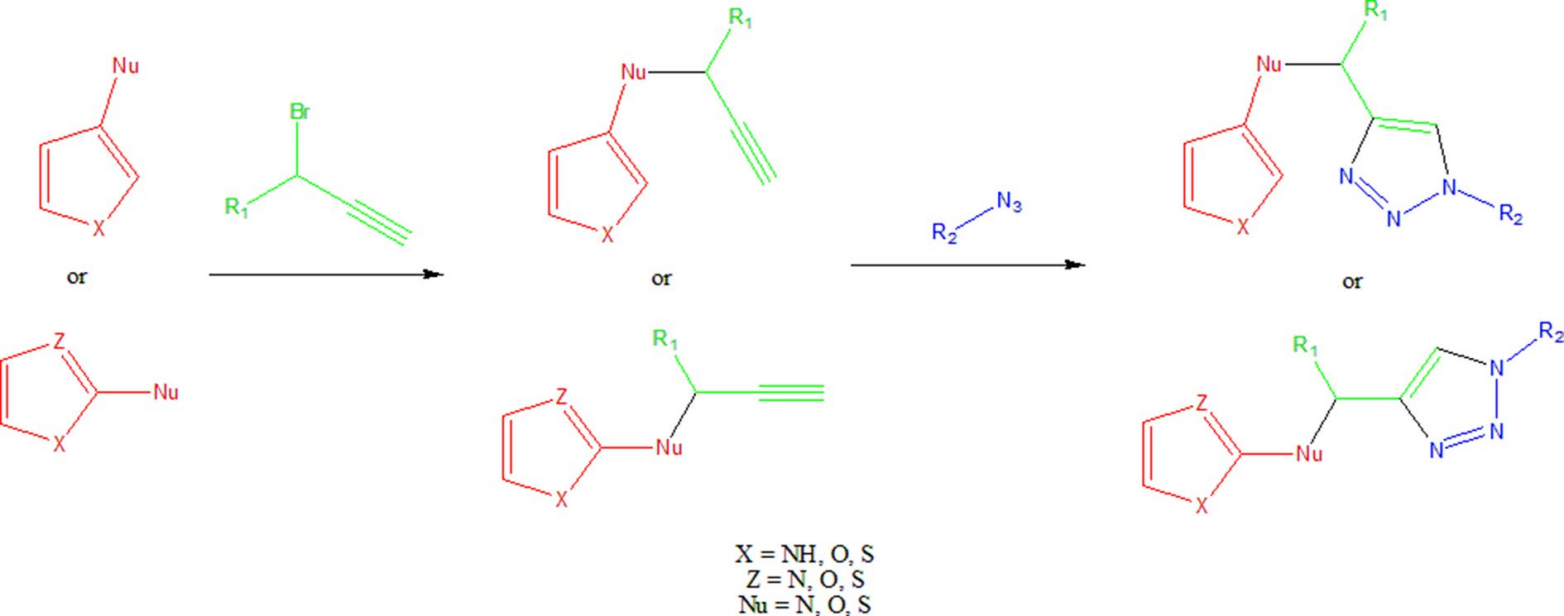
A strategy used to build bis-heterocycles


This example is based on the synthetic approach reported by Shafi et al. [79] to obtain *bis*-heterocycles, linking 5-membered heterocycles building blocks containing one or two heteroatoms (at least one nitrogen, sulfur, oxygen) to a set of azide containing building blocks through the formation of a 1,4-disubstituted 1,2,3-triazole using click chemistry (Fig. 3). To this purpose, the heterocycle must contain a nucleophilic moiety such as a thiol, hydroxyl, or amino group that reacts with a 3-halopropyne derivative through nucleophilic aliphatic substitution (S_*N*_). Once the alkyne is appropriately attached to the heterocycle, it reacts with the set of azides to form a 1,2,3-triazole linking both fragments. Python and the chemoinformatics toolkit RDKit [23] are used to implement algorithms and functions in this example. The toolkit RDKit provides the capabilities to handle and manipulate molecular structures in Python. A comprehensive introduction and installation instructions can be found in the online documentation from the RDKit homepage (https://rdkit.org/docs/index.html).

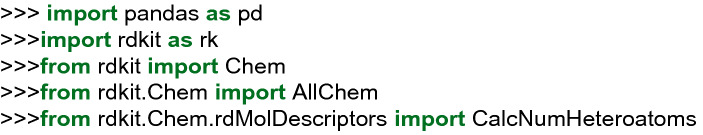

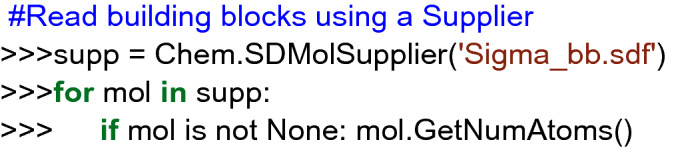

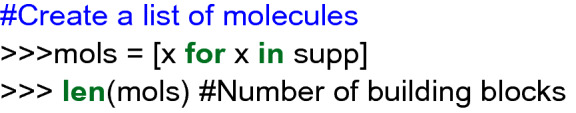

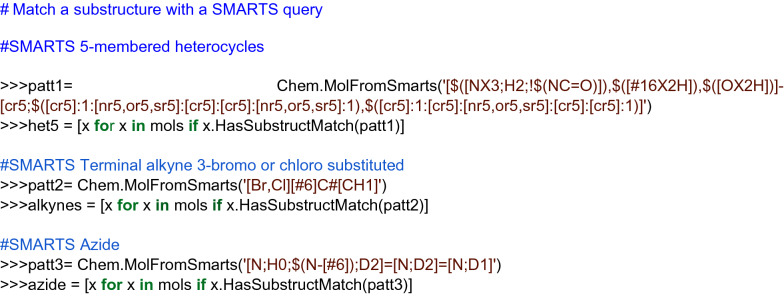


Procedure in Python:

1. Build or identify a library of commercially available building blocks. The building blocks used for this example were taken from the Sigma Aldrich (Building Blocks) catalog obtained from the ZINC DB [80], consisting of 124,368 building blocks.
2. Identify the characteristics of building blocks for the strategy to be followed. Minor components and duplicate compounds were removed, building blocks were selected to comply with the Congreve’s ‘rule of three’ [71]. The curated database can be found in Additional file 1: *"Sigma_bb.sdf."* As shown below, the building blocks were read in Python using a supplier. Then, compounds were filtered for the presence of appropriate functional groups: a 5-membered heterocyclic ring with one (N, O or S) or two heteroatoms (N, O, S; at least one N), and a nucleophilic substituent (–OH, –SH, –NH_2_), a terminal alkyne 3-bromo or chloro substituted and an azide.
3. Setting up coupling reactions. To generate the library of bis-heterocycles, the reactions and their corresponding SMIRKS were defined according to a synthetic approach reported by Shafi et al. [79] (Table 5). These reactions were used in the code to enumerate compounds that were eventually exported in CSV format.
4. Results. In total, 7884 bis-heterocycles were obtained. Examples of compounds obtained following this strategy and using the Sigma Aldrich building block database are shown in Table 9.

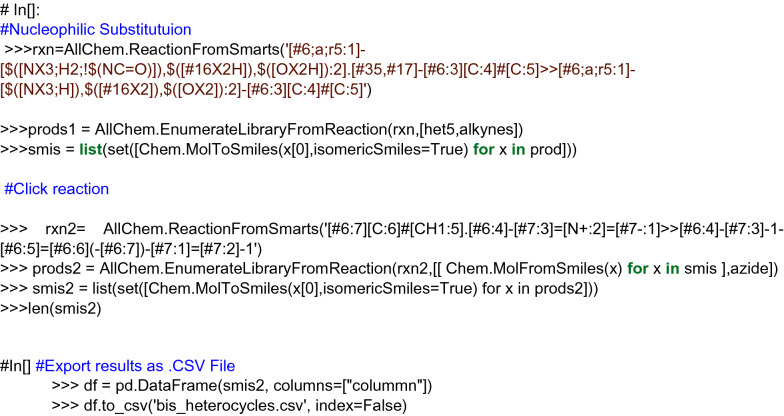

**Table 5:**
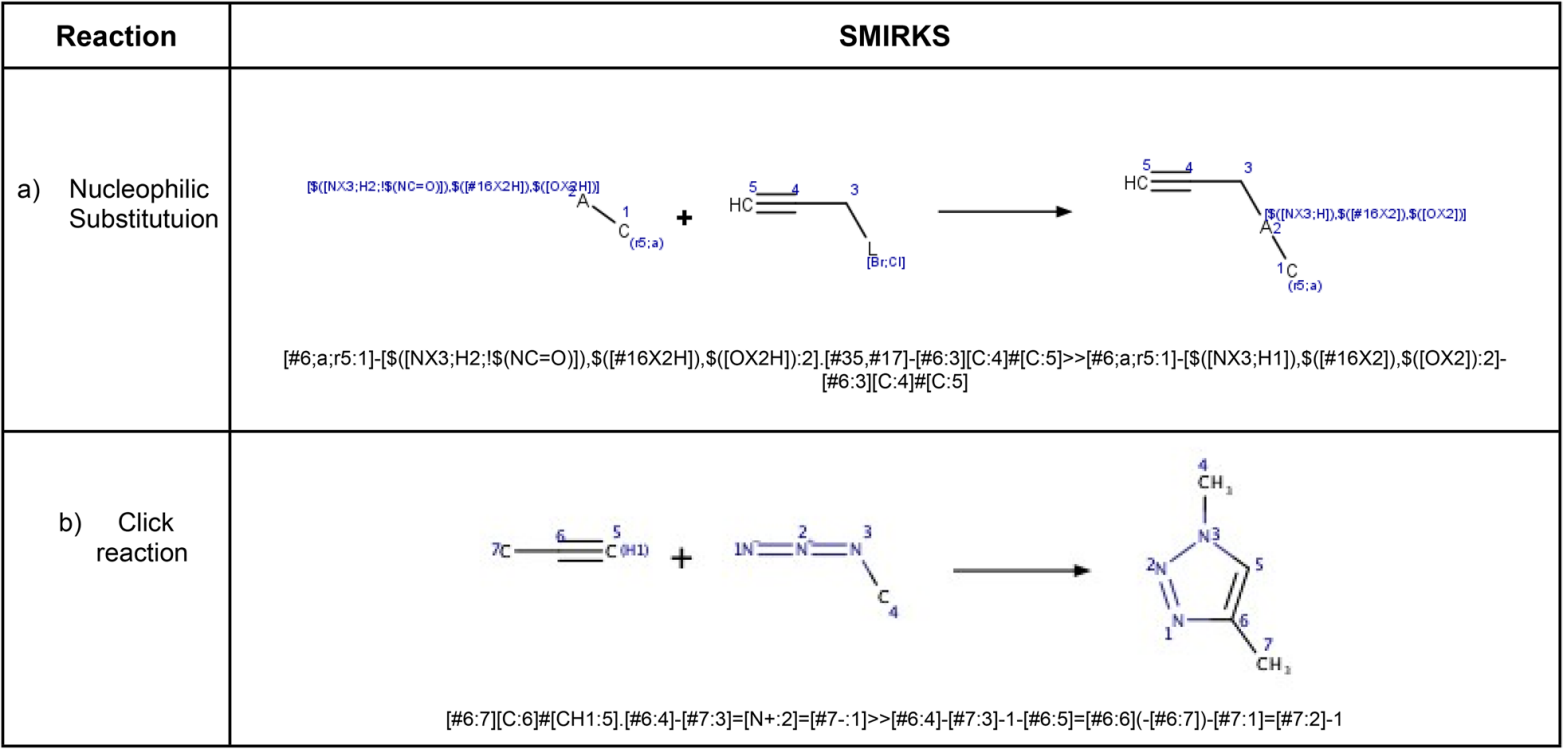
SMIRKS of the coupling reactions

## Design of a DOS library using KNIME and RDKit and Marvin nodes

Lactams are a class of compounds important for drug design due to their great variety of potential therapeutic applications, spanning from cancer [81, 82], diabetes [83], and infectious diseases [84]. Many lactam-containing compounds are reported to act as HIV-1 integrase inhibitors [85], opioid receptor agonists [86, 87], as well as antitumoral [88, 89], anti-inflammatory [90, 91], and antidepressant agents [92]. For the first example, a library of lactams was automated by applying the DOS strategy Build/Couple/Pair [93] for medicinal chemistry applications [94]. The Build/Couple/Pair approach consists of building different starting materials with suitable functional groups that can be joined together through intermolecular coupling reactions in all possible stereochemical combinations. In the pairing step, intramolecular coupling reactions that join the remaining functional groups are instrumental for developing skeletal diversity and structurally different molecular scaffolds. The KNIME (Konstanz Information Miner) workspace [20] was selected as a platform for generating the workflow, where each task is represented by a node with input and output ports. This server can be downloaded directly from the KNIME homepage (https://www.knime.com/). For the management and analysis of databases, the KNIME Example Server provides access to many explanatory workflows. The example server is accessible via the KNIME Explorer panel within the KNIME workbench and represents a great help when starting a new workflow.

**Fig. 4:**
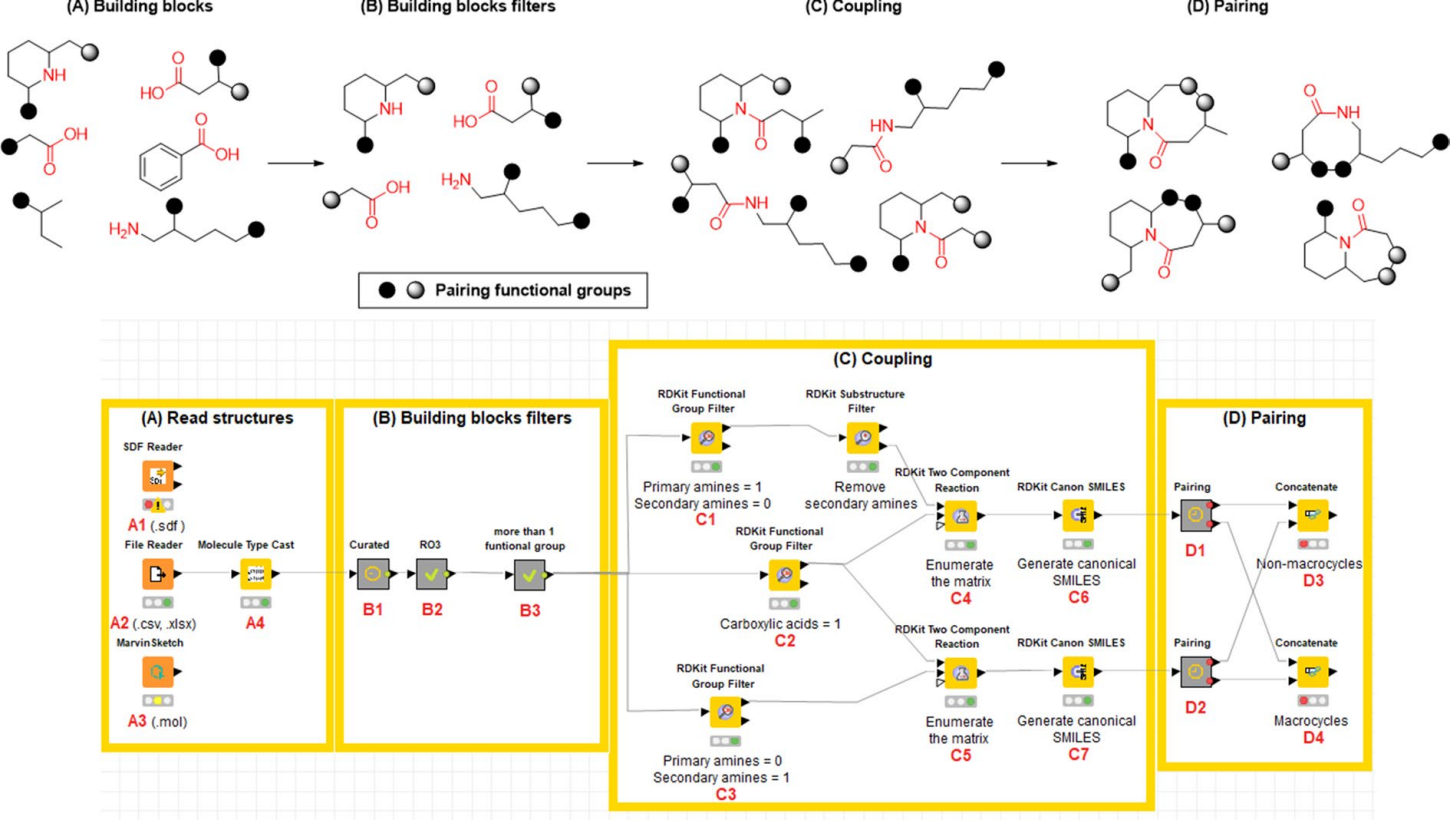
Workflow for the design of lactams. **a** Read structures of building blocks; **b** Building blocks filter: the structures were curated, filtered according to the ‘rule of three’, and selected for the presence of appropriate functional groups; **c** Coupling phase: application of the amide bond formation reaction between carboxylic acids and primary or secondary amines; **d** Pairing phase: use of the reactions as described in Table 8. Finally, the compounds were separated into macrocycles and not macrocycles

Figure 4 shows the workflow designed to generate a library of lactams following the B/C/P approach. The development of this workflow is described in detail below.

1. Build or identify a library of commercially available building blocks. We selected the commercially Enamine building blocks library as a first input for this tutorial, containing 437,625 unique compounds (version March 2019) [95]. To allow for the readability of all datasets, nodes for retrieving molecules in different formats were considered, including the SDF file (structure data file) (A1) or CSV file (comma-separated value) (A2). The Marvin Sketch node (A3) was also included to draw other possible building blocks.
2. Identify the characteristics of building blocks for the strategy to be followed. Compounds were normalized, minor components and duplicate compounds were removed (B1), building blocks were selected in to comply with the Congreve’s ‘rule of three’ [71] (B2), and then filtered for the presence of appropriate functional groups (B3). The strategy used required building blocks with more than two functional groups: one for the coupling reaction and another for the pairing reaction. The functional groups used in this part and their corresponding SMARTS codes are listed in Table 6.

**Table 6:** Functional groups that were quantified to filter building blocks

Functional groups	SMARTS
Alkene	[H]\[#6]([H]) = [#6]/[#6]
Alkyne	[H]C#C[#6]
Carboxylic Acid	C(= O)[O;H,-]
Sulfonyl chloride	[$(S-!@[#6])](= O)(= O)(Cl)
Amine primary	[N;H2;D1;$(N-!@[#6]);!$(N–C = [O,N,S])]
Amine secondary	[N;H1;D2;$(N(-[#6])-[#6]);!$(N-[!#6;!#1]);!$(N–C = [O,N,S])]
Alcohol aromatic	[O;H1;$(O-!@c)]
Alcohol aliphatic	[O;H1;$(O-!@[C;!$(C = !@[O,N,S])])]
Aldehyde	[CH;D2;!$(C-[!#6;!#1])] = O
Halogen	[$([F,Cl,Br,I]-!@[#6]);!$([F,Cl,Br,I]-!@C-!@[F,Cl,Br,I]);!$([F,Cl,Br,I]-[C,S](= [D1;O,S,N]))]
Azide	[N;H0;$(N-[#6]);D2] = [N;D2] = [N;D1]
3. Setting up coupling reactions. To generate a library of lactams, only the amide bond formation between carboxylic acids (C2) and primary (C1) or secondary amines (C3) was considered as the coupling reaction (C4 and C5), the SMIRKS of this reaction is showed in Table 7. The SMILES of both secondary and tertiary amides-containing coupling products were generated (C6–C7).

**Table 7:**
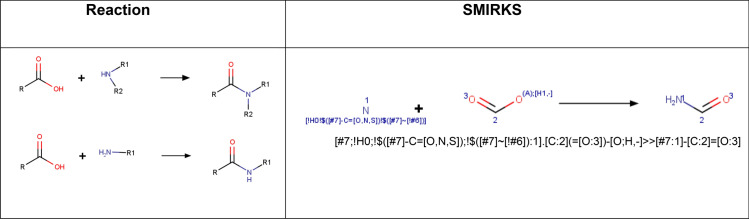
SMIRKS of the amide bond formation between carboxylic acids and primary or secondary amines
4. Establish pairing reactions. Then different intramolecular cyclization reactions were applied for the pairing phase (D1–D2). Compounds containing the two functional groups involved in the pairing reaction within the same building block were removed. This step was done to ensure that the lactam-containing ring was closed. Table 8 shows the different intramolecular cyclization considered for the pairing phase and their corresponding SMIRKS.

**Table 8:**
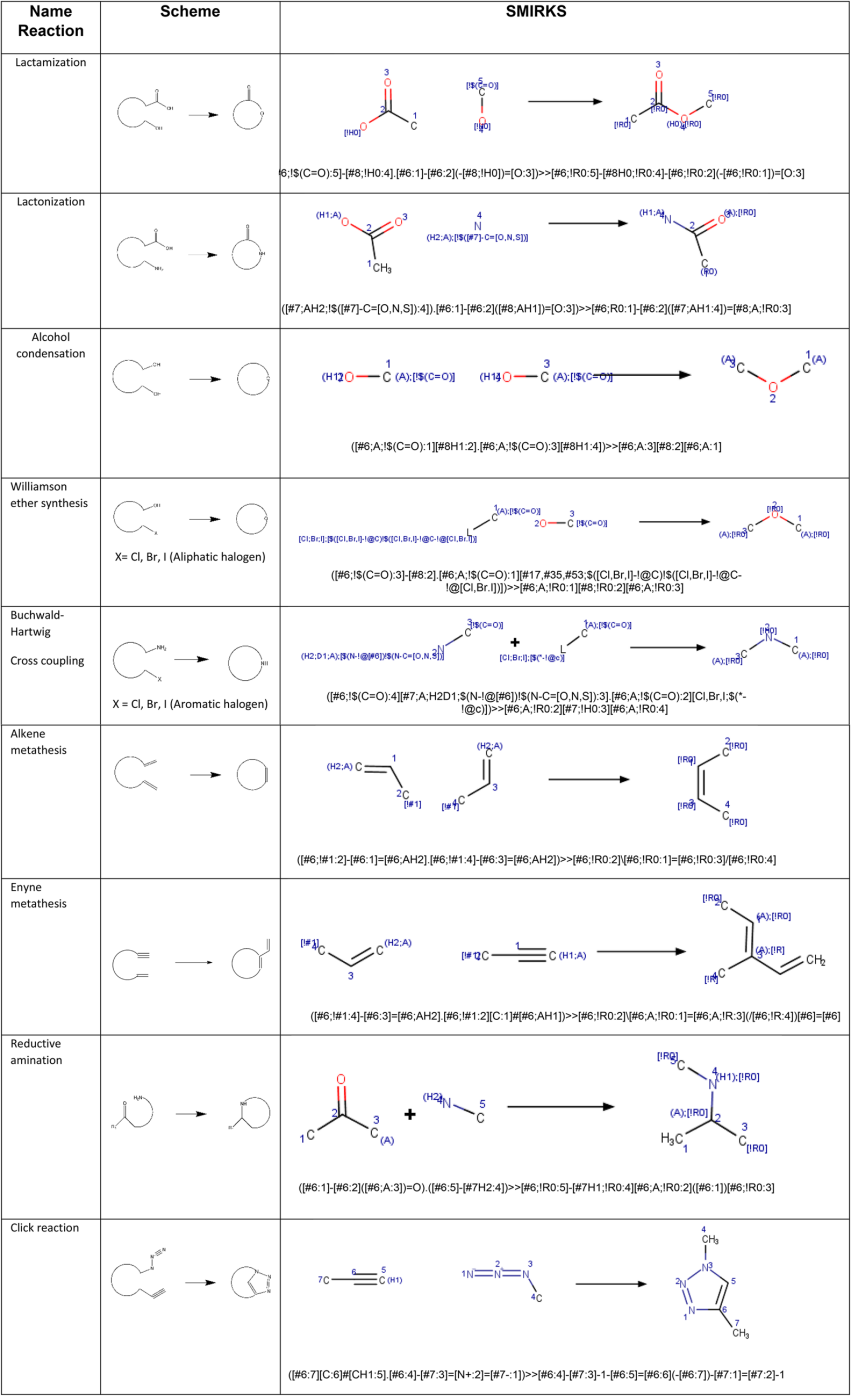
Intramolecular cyclization considered for the pairing phase
5. Separated into macrocycles and not macrocycles. The lactams obtained from the DOS B/C/P workflow were divided into macrocycles (more than 7-membered rings) and non-macrocycles (3- to 7-membered rings). Examples of non-macrocyclic lactams that were produced under this approach are shown in Table 9. Information about the number of compounds generated and the database’s diversity was published by Saldivar-González et al. [94].

## Library of isoindolinone based compounds as potential AChE inhibitors

Alzheimer’s disease (AD) is an incurable, progressive neurodegenerative disorder with a long presymptomatic period. It is clinically characterized by cognitive and behavioral impairment, social and occupational dysfunction and, ultimately, death [96]. The enhancement of cholinergic neurotransmission by preserving acetylcholine (ACh) levels would be an effective way to overcome AD’s occurrence, symptoms, and progression. Accordingly, the inhibition of acetylcholinesterase (AChE), which is responsible for the metabolic breakdown of ACh has been regarded as one of the most promising approaches [97]. Although various efficient cholinesterase inhibitor drugs such as donepezil, rivastigmine, and galanthamine have been developed, there is still significant demand for drug discovery leading to efficient anti-Alzheimer’s agents [98].

Isoindolinones are an important heterocyclic scaffold ubiquitous in natural products such as aristoyagonine, nuevamine, lennoxamine, and chilenine [99]. Recently, Rayatzadeh et al. [98] reported the synthesis and acetylcholinesterase inhibitory activity of novel isoindolinone derivatives, in which two of the tested compounds showed an IC_50_ of 41 and 83 μM, respectively. Even more, the compounds were obtained through a convenient procedure in the absence of any catalysts or additives in an Ugi reaction with good tolerance to diverse functional groups and satisfactory yields between 70 and 90%. This background information attracted our attention, so we decided to use the approach reported to be an example of how a library can be built with an established scaffold and a targeted biological activity.

Data Warrior was selected as a platform for the generation of this example. This software is a universal data analysis and visualization program, useful to explore large data sets of chemical structures with alphanumerical properties [19]. Some of its functionalities include combinatorial library enumeration, the prediction of molecular properties, and various methods to visualize chemical space and activity cliffs with the intent to support chemists taking smarter decisions about structural changes toward better property profiles.

**Fig. 5:**
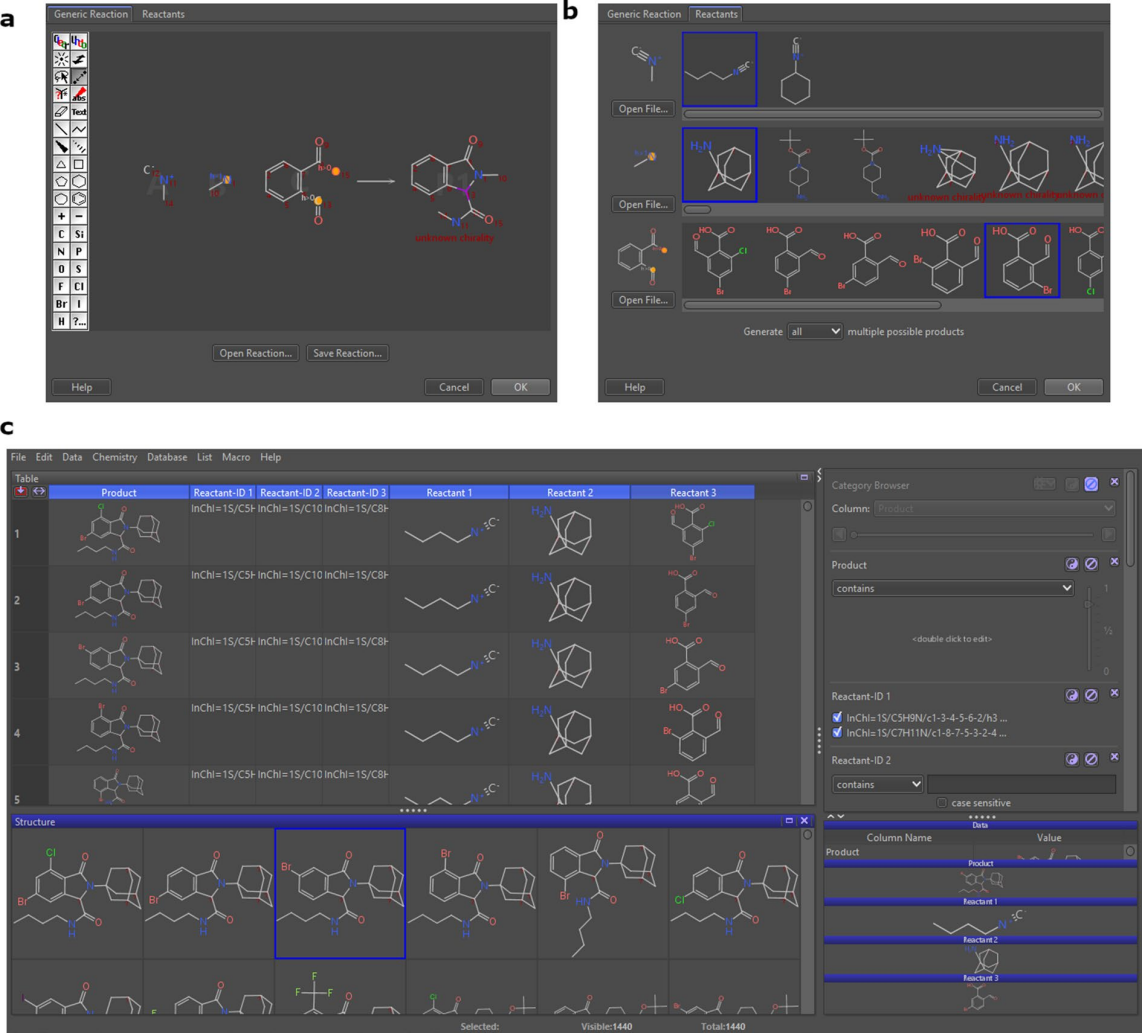
**a** Reaction input tab in Enumeration of Combinatorial Library; **b** Reactants input tab in Enumeration of Combinatorial Library; **c** View of the library generated

Procedure in Data Warrior:

1. Build or identify a library of commercially available building blocks. For this example, building blocks' primary input was the Synquest Building Blocks Economical catalog retrieved from the ZINC DB [100], consisting of 59,597 building blocks. However, derivatives of 2-carboxybenzaldehyde were not found in this database, so a SMARTS containing the moiety was used to search for building blocks directly in all ZINC DB catalogs [101]. The screenshots and steps of how this search was performed can be found in Additional file 1.
2. Identify the characteristics of building blocks for the strategy to be followed. Minor components and duplicate compounds were removed using Bank-Cleaner server (https://mobyle.rpbs.univ-paris-diderot.fr/cgi-bin/portal.py?form=FAF-Drugs4#forms::Bank-Cleaner), then building blocks were selected to comply with the Congreve’s ‘rule of three’[71] with the filter parameters created at the FAF-Drugs4′s Filter Editor (https://mobyle.rpbs.univ-paris-diderot.fr/cgi-bin/portal.py?form=Filter-Editor#forms::Filter-Editor), and running the filter at FAF-Drugs4′s Filtering Tool (https://mobyle.rpbs.univ-paris-diderot.fr/cgi-bin/portal.py?form=FAF-Drugs4#forms::FAF-Drugs4). The filter parameters can be found in Additional file 1. The functional groups needed were filtered using the Data Warrior substructure search. The detailed procedure and the substructures defined to filter can be found in Additional file 1 (“Substructure filtering in Data Warrior” section). In this case, the three-component Ugi reaction required an isocyanide and a primary amine, which were obtained from the Synquest Building Blocks, and 2-carboxybenzaldehyde, obtained from the ZINC catalog. Additionally, to include only groups that would add flexibility to the final compound, for isocyanides and primary amines, the building blocks containing aromatic rings were eliminated.
3. Establish the three-component reaction. Using the Create Combinatorial Library on the Chemistry module of Data Warrior, the reaction was built in its simpler form under “Generic Reaction,” only drawing the atoms involved in the transformation and adequately mapping each atom from the reagents into its position in the product (Fig. 5a). An.RXN file with the reaction already drawn in another program can also be imported. The list of building blocks previously created for each of the reactants in.SDF format was imported (Fig. 5b), and the library was generated.
4. Results. The SMILES of the isoindolinones were obtained, generating 738 different compounds. Examples of isoindolinones that were generated under this approach are shown in Table 9.

**Table 9:**
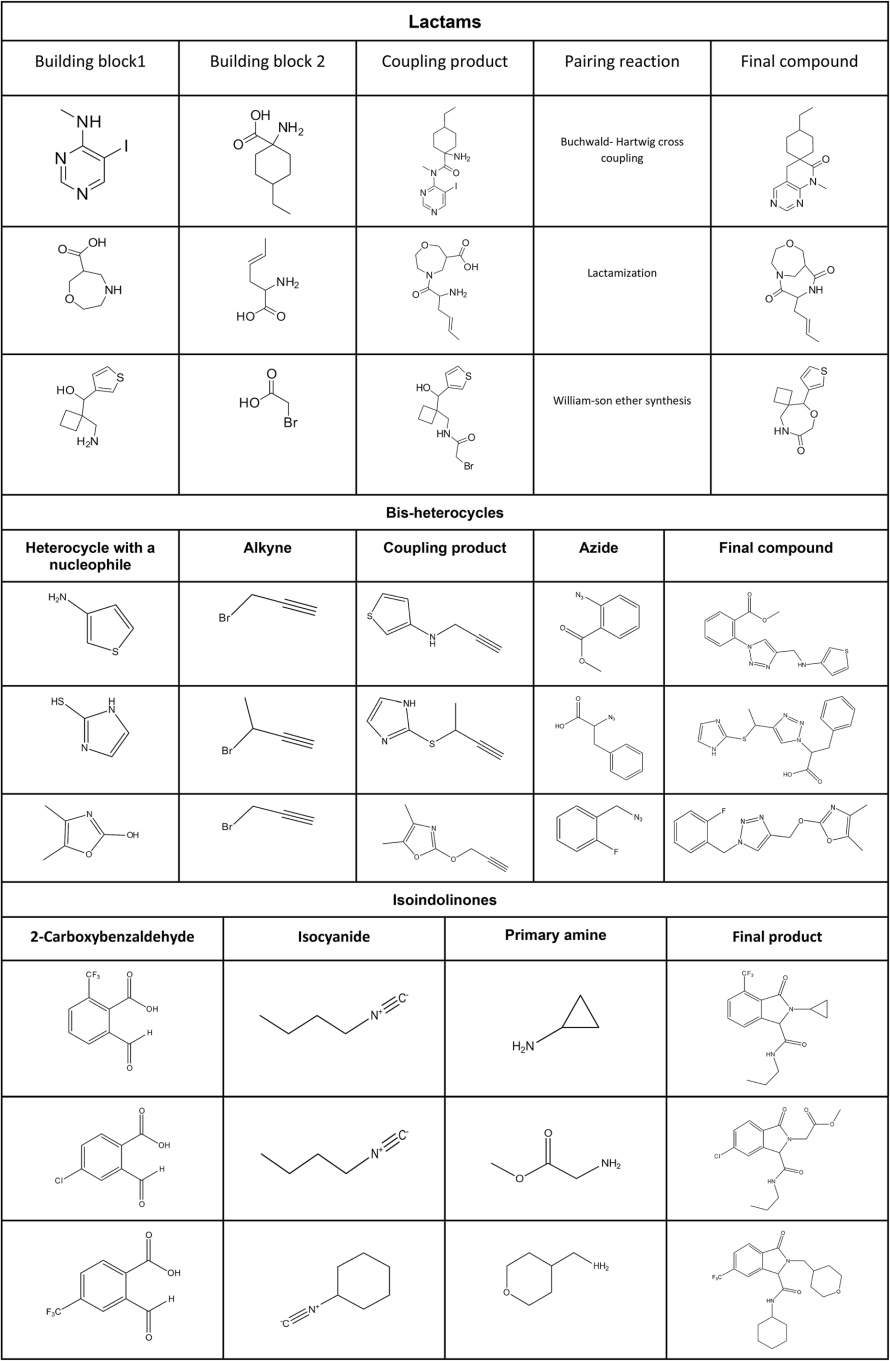
Representative examples of compounds from the three libraries design in this work

## Post-processing virtual libraries

### Diversity analysis

Before performing a virtual screening or the synthesis of a virtual compound, it is convenient to characterize the compounds generated using different criteria. For example, profiling the compound library with whole molecule descriptors of pharmaceutical relevance can help to validate the strategy used, represent medicinally relevant chemical spaces [102], and filter compounds with drug-like properties [103, 104]. Physicochemical properties frequently used to describe chemical libraries include molecular weight (MW), number of rotatable bonds (RBs), hydrogen-bond acceptors (HBAs), hydrogen-bond donors (HBDs), topological polar surface area (TPSA), and the octanol/water partition coefficient (SlogP).

A complementary approach to characterize compound databases is through molecular scaffolds or chemotypes i.e., a molecule’s core structure [105]. Scaffold analysis is broadly used to compare compound databases, to identify novel scaffolds in a compound library, to measure diversity based on molecular scaffolds [106], to evaluate the performance of virtual screening approaches [107], and to analyze the SAR of sets of molecules with measured activity [108–110]. Like physicochemical properties, molecular scaffolds are easy to interpret and facilitate communication with a scientist working in different disciplines. Another approach, perhaps more difficult to interpret but widely used to characterize databases and has been successfully applied to a series of computer-assisted chemoinformatics and drug design applications, is the molecular fingerprints [111]. Fingerprints are especially useful for similarity calculations, such as database searching or clustering, generally measuring similarity as the Tanimoto coefficient [112].

In addition to helping in the characterization of databases, these chemoinformatic approaches are useful for determining the chemical and structural diversity of the compounds generated. The quantitative information generated helps guide the selection of compound libraries or individual compounds to identify novel lead candidates for biological targets. In particular, diversity analysis helps compare different databases and evaluate the structural novelty of a compound collection [113]. Free tools such as RDKit [23], Platform for Unified Molecular Analysis (PUMA) [114], or the workflows developed in KNIME by Naveja et al. [115] can help in the task of assessing chemical diversity. Interpreting the results of these analyzes individually, in many cases, is complicated and can lead to biased interpretations since, as previously mentioned, the perception and evaluation of the diversity of a collection of compounds, in general, is relative to the molecular representation. To decrease the diversity’s dependence with molecular representation, it has been proposed to use a consensus approach through the assessment of global diversity using Consensus Diversity Plots (CDPs). A CDP is a 2D graph that represents in the same plot up to four measures of diversity. The most common are fingerprint-based, scaffold, whole molecular properties associated with drug-like characteristics, and database’s size [116].

**Fig. 6:**
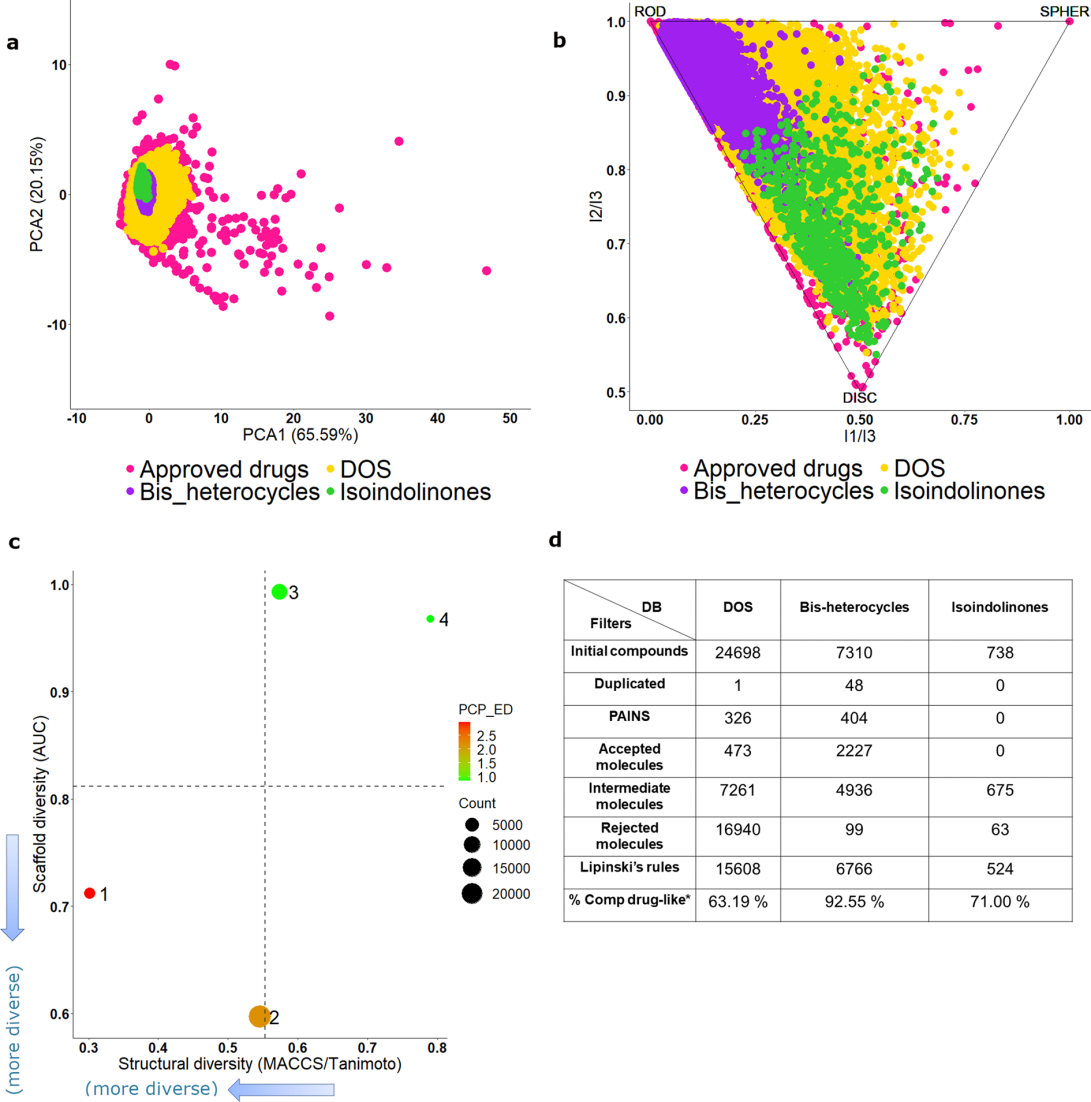
Post-processing plots. **a** PCA plot generated using six structural and physicochemical descriptors (MW, HBA, HBD, SlogP, TPSA and RBs). **b** PMI plot. Compounds are placed in a triangle where the vertices represent rod, disc, and spherical compounds. **c** Consensus Diversity Plot (CDP): (1) Approved drugs, (2) DOS, (3) Bis-heterocycles, (4) Isoindolinones. Scaffold diversity is measured in the vertical axis using area under the curve (AUC) and the diversity using molecular fingerprints is measured in the horizontal axis using MACCS/Tanimoto. Diversity based on physicochemical properties is represented by the Euclidean distance of the six physicochemical properties using a continuous color scale. The relative size of the data set is represented by the size of the data point. **d** ADME/Tox profile of the three databases calculated with the free server FAF-Drugs. *Based on Lipinski’s Rule of Five

For the three compound libraries designed in this manuscript (lactams, bis-heterocycles, and isoindolinones), their chemical space based on physicochemical properties and shapes was analyzed and compared with a reference library of approved drugs. Their global diversity of each database was also analyzed using the CDPlot. Figure 6a illustrates an application of PCA to generate a visual representation of the property-based chemical space of 24,698 lactams,7884 bis-heterocycles, 649 isoindolinones, and a collection of 2125 drugs approved for clinical use obtained from DrugBank [117]. PCA is a mathematical method for dimensionality reduction that allows us to visualize similarities and differences within collections of compounds based on structural and physicochemical parameters [118], making it a valuable tool to guide the design of chemical libraries. The figure shows that the three libraries designed in this manuscript occupy the same property space as the main part of the approved drugs library, indicating that the compounds are prone to have favorable drug-like properties. Out of the three design libraries, the DOS collection is the most diverse, covering almost the same space as approved drugs. In contrast, the bis-heterocycles and isoindolinones are less diverse and focus on a region of the space. Because of the design strategy, the property space of bis-heterocycles’ library space is more restricted to the heterocycles and azides. Since the isoindolinones library was designed based on a common scaffold, the variations of the molecular properties depend on the side-chain substitutions. Thus, it is not surprising that they are focused on a more restricted region in chemical space.

The molecular shape is also a useful property to define chemical spaces [119]. In the PMI plot in Fig. 6b, we can see that the main space occupied by approved drugs is between rod and disc shapes, and once again, we can observe the three libraries designed to share that space. Bis-heterocycles and isoindolinones libraries are focused in a specific shape. On one side, bis-heterocycles are predominantly in the PMI plot’s disc zone because the azide and heterocyclic fragments were linked, forming a 1,4-disubstituted 1,2,3-triazole in the middle, obtaining large molecules. Furthermore, two aromatic rings highly restricted the flexibility of the fragments linked, forcing the molecule to be in an extended position (Table 9). On the other side, isoindolinones are mainly in the disc zone of the PMI plot because the scaffold ring is planar so that the main shape variations will be caused only by the substituents in positions 1 and 2 of the ring (Table 9). Some substituents at position 2 of isoindolinones could cause the molecules to grow in a rod shape, explaining why a few molecules of this library tend to expand into the rod zone. Similarly, the planarity of bis-heterocycles explains that fewer compounds in this library grow into the ring space. DOS library is centered in the shape space, similar to approved drugs, because of its larger structural diversity. In contrast to the other two libraries designed in this work, compounds in DOS explore the sphere zone with potentially drug-like properties.

Figure 6c shows the CDP of the libraries designed in this work. The size of the data points represents the relative size of each data set, and the color of each data point represents the diversity of the physicochemical properties of the data set as measured by the Euclidean distance of six properties of pharmaceutical relevance (MW, HBAs, HBDs, TPSA, SlogP, RBs). To measure the structural diversity considering the entire structures (including not only the central scaffold but also the side chains) (x-axis), the MACCS fingerprints were used, and then the Tanimoto coefficient was applied [120]. Values outside the similarity matrix’s diagonal were used to compute the median for all the pairwise comparisons. On the other hand, as a measure of scaffold diversity, the Area Under the cyclic system recovery Curve (AUC, y-axis) [121] was used. Scaffolds were generated under the Bemis-Murcko definition [122]. The AUC value is a useful parameter to evaluate the diversity of the scaffold’s content in each database. AUC value ranges from 0.5 (maximum diversity, when each compound in the library has a different cyclic system) to 1.0 (minimum diversity, when a single cyclic system encompasses all the compounds). According to Fig. 6c, the DOS library is the most diverse of all three designed libraries when considering all three diversity criteria: high scaffold and physicochemical diversity, and intermediate fingerprint diversity. Approved drugs are also very diverse when considering scaffold and fingerprints; however, the variety in physicochemical properties is lower. The relative lower scaffold diversity of bis-heterocycles and isoindolinones (with an area under the scaffold recovery curve, AUC, close to one—Fig. 6c) agrees with the design strategy of both libraries that is focused on the scaffolds. In bis-heterocycles, without considering the heterocycle, the structural variation associated with the azides is more considerable, causing larger fingerprint-based diversity than isoindolinones. In isoindolinones, even if the number of different amines and isocyanides is limited, the three-component reaction (described in section “Library of isoindolinone based compounds as possible AChE inhibitors”, vide supra) offers a larger amount of combinations, increasing the physicochemical diversity.

However, it is vital to keep in mind that even in the design and synthesis of focused libraries, there must be some degree of diversity, and "redundant" compounds (molecules that are structurally similar and have the same activity) should be avoided. A diverse subset of compounds should be more likely to contain compounds with different activities and should also contain fewer "redundant" compounds. For this reason, the metrics used above can also be useful for navigating through the relevant chemical space to identify subsets of compounds for synthesis, purchase, or testing. Approaches to select subsets efficiently are mainly cluster analysis, dissimilarity-based methods, cell-based methods and optimization techniques [123]. If you want to repeat this study, you can use the file titled "Diversity Analysis.csv" and use the PUMA server (https://www.difacquim.com/d-tools/) or the workflows reported by Naveja et al. [115].

## ADME/Tox profile

Other than the diversity analysis described in the previous section, in order to reduce the number of compounds to be used in virtual screening, filters like functional groups, physio-chemical properties, PAINS, and toxicophores can be applied using free servers like FAF-Drugs (https://mobyle.rpbs.univ-paris-diderot.fr), Chembioserver 2.0 (https://chembioserver.vi-seem.eu/index.php) and the workflows designed in KNIME [124–126].

The compounds of three libraries obtained in this work were analyzed in FAF-Drugs to filter undesirable compounds and assist hit selection before chemical synthesis. In this server, depending on the filtering ranges, Accepted (compounds with no structural alerts and satisfying the physicochemical filter), Intermediate (compounds which embed low-risk structural alerts with several occurrences below the threshold) or Rejected (compounds that include a high-risk structural alert) files are written associated with all their CSV results files [127]. According to the FAF-Drugs results, it can be seen in Fig. 6d that the compounds identified as bis-heterocycles have more drug-like physicochemical properties; however, it is the isoindolinone database that contains the fewest structural alerts. In contrast, the database of lactams obtained by the B/C/P DOS strategy is the one that contains the largest amount of PAINS and rejected molecules. The main problematic moieties in this database are shown in Additional file 1: Figure S1, where many fluorenylmethyloxycarbonyl compounds are associated with promiscuity [128], and compounds with an excess of halogens in their structure are observed.

## Synthetic accessibility

The number of designed compounds in silico may still be vast, and some of them may not be easy to synthesize in the laboratory. Therefore, an estimate of the synthetic accessibility, or, make filters related to reagents’s cost, in principle, could help filter further the database or prioritize the structures generated.

If an approach based on known reaction schemes was not applied, it would be necessary to evaluate the synthetic feasibility of the possible synthetic routes. The optimal method for evaluating a given compounds’ synthetic feasibility is probably to search the chemical literature for cases where this or similar molecules/scaffolds have been synthesized and to check the results with experienced organic chemists [13]. Some of the tools available for planning synthetic routes are SciFinder [129], Reaxys [60], Synthia [130], spaya.ai [131], and IBM RXN [132], of which the last two mentioned are open access; being an area of research growing in parallel with the technologies available, we should always keep an eye on developing tools such as AutoSynRoute [133] and new evaluation methods [134]. Unfortunately, this is not an accessible approach in an automated algorithm to filter the input to a large-scale virtual library, so computer-based methods to evaluate synthetic accessibility have been developed.

Synthetic accessibility is related to the ease of synthesis of compounds according to their synthetic complexity, which combines starting materials information and structural complexity [135], and is usually measured through a score (SAscore) on a determined scale. Different tools are available to measure the synthetic accessibility of molecules. Some examples are SYLVIA [136], CAESA [137], WODCA [138], an RDKit Python source [139], an scoring function in C +  + based on the MOSES software library [140], as well as other methods reported [141].

## Conclusions

In recent years, the generation of virtual libraries has had unprecedented progress thanks to the development of different computational methods and synthetic knowledge. Virtual libraries represent an important source of novel structures in drug discovery applications. This work showed how, through different computational open-access methods, it is possible to automate design approaches and enumerate and explore all the compounds obtained using pre-validated reactions and commercially or in-house available building blocks. These methods are becoming increasingly sophisticated and allow restrictions on compound synthesis and filters to prevent the creation of unwanted chemical compounds. The importance of the post-processing step should always be remembered, bearing in mind that the aims of generating virtual libraries should be focused on generating molecules that are more attractive to medicinal chemists, both improving the quality of compounds manufactured and making sure they are synthetically accessible. We have shown how different previously reported tools and software available can be used on the generated libraries to predict critical pharmacological properties, molecular shape or to compare them to already existing libraries.

The tutorial examples used in this manuscript show that it is possible to generate libraries with predicted drug-like properties using validated reactions and commercially available building blocks. Some of the generated compounds explore novel areas of the molecular shape space, compared to approved drugs. We are confident that the approaches used in this manuscript will flourish (hopefully, with the aid of this tutorial), as long as the knowledge derived from organic synthesis continues to be captured and exploited. We also anticipate that more academic groups will use these strategies to design new chemical structures.

## Supplementary information

**Additional file 1.** This document describes the substructure search in the ZINC database; the filter parameters for Congreve’s Rule of 3 used in the FAF-Drugs server; the instructions for filtering substructures in Data Warrior and Figure S1.

## Data Availability

Data and materials for the examples are available as additional materials. For Example 1 “Bis-heterocycles” the curated database of building blocks can be found as “Sigma_bb.sdf”, the python code as “BisHet.py” and the library generated can be found as “bis-heterocycles.csv”. For Example 2 “DOS” the building blocks were retrieved from the ZINC DB catalogs as previously described, the KNIME workflow used is “Workflow_DOS.knwf” and the library generated can be found as “LactamsDOS.csv”. For Example 3 “Isoindolinones” the building blocks were retrieved from the ZINC DB catalogs as previously described, the input file used in Data Warrior in SDF format are included as: “synquestecbb.sdf” and “2-carboxybenzaldehydes.sdf”. The reaction file is “Ugi-3comp.rxn”. And finally, the library generated can be found as “Isoindolinones.sdf”. The compounds from the three libraries generated in this work and the drugs approved used for the diversity analysis can be found as "Diversity Analysis.csv".

## Abbreviations

AChE: Acetylcholinesterase
AD: Alzheimer disease
CSV: Comma separated value file
CDP: Consensus Diversity Plot
DOS: Diversity-Oriented-Synthesis
HBAs: Hydrogen-bond acceptors
HBDs: Hydrogen-bond donors
InChi: IUPAC International Chemical Identifier
InChIKey: A fixed-length (27-character) condensed digital representation of an InChI
KNIME: Konstanz Information Miner
MW: Molecular weight
RBs: Number of rotatable bonds
SA: Synthetic accessibility
SAR: Structure–activity relationship
SDF: Standard data file
SLogP: Octanol/water partition coefficient
SMARTS: SMILES Arbitrary Target Specification
SMILES: Simplified Molecular Input Line System
SMIRKS: Language to define generic reactions. It is a hybrid of SMILES and SMARTS languages
PAINS: Pan Assay Interference Compounds
PMI: Principal Moment of Inertia
PUMA: Platform for Unified Molecular Analysis
TOS: Target-Oriented Synthesis
TPSA: Topological Polar Surface Area
