# Supplementary material for: Chemoinformatics-based enumeration of chemical libraries: a tutorial
Source: J Cheminform. 2020 Oct 27;12:64. doi: 10.1186/s13321-020-00466-z (PMC7590480; doi:10.1186/s13321-020-00466-z)
Supplement: Supplementary file 1 — Additional file 1. This document describes the substructure search in the ZINC database; the filter parameters for Congreve’s Rule of 3 used in the FAF-Drugs server; the instructions for filtering substructures in Data Warrior and Figure S1. [file 13321_2020_466_MOESM1_ESM.zip › Additional file 1/Additional file 1.pdf]

Chemoinformatics-based enumeration of chemical libraries: A tutorial

Fernanda I. Saldívar-González[a*], C. Sebastian Huerta-García[b], José L. Medina-Franco[a]

[a] DIFACQUIM research group, School of Chemistry, Department of Pharmacy, Universidad Nacional Autónoma de México, Avenida Universidad 3000, Mexico City 04510, Mexico

[b] School of Chemistry, Department of Pharmacy, Universidad Nacional Autónoma de México, Avenida Universidad 3000, Mexico City 04510, Mexico

*Corresponding author: fer.saldivarg@gmail.com

CONTENTS

ZINC DB catalog search by substructure S2

Filter parameters for Congreve's Rule of 3 used in FAF-Drugs server S4

Substructure filtering in Data Warrior S5

Figure S1. Results obtain in FAF-Drugs S10

1) ZINC DB catalog search by substructure

a) Go to https://zinc.docking.org/ and click on Substances

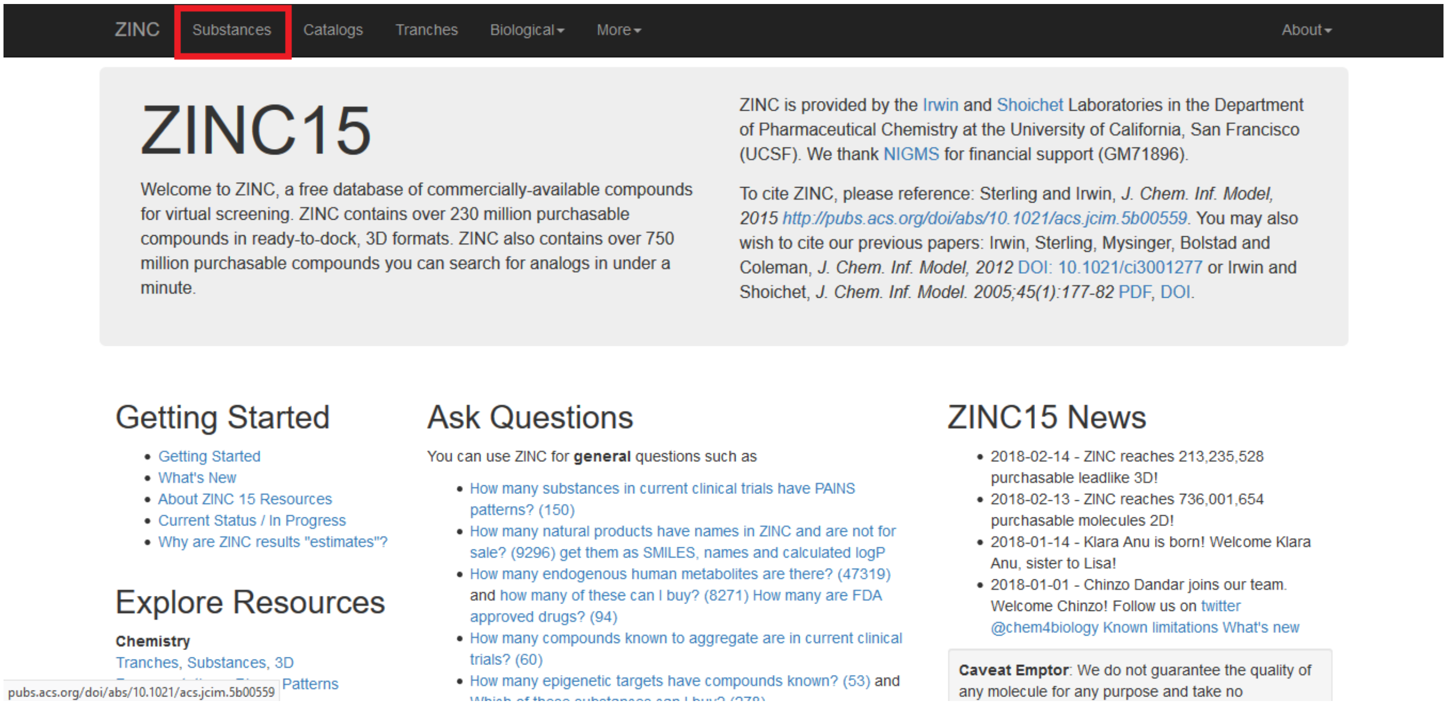

b) In the "Search Using One" section, the following SMARTS was used: [H][#6](=O)-[#6]-1=[#6]-[#6]=[#6]-[#6]=[#6]-1-[#6](-[#8])=O (1). The Building Blocks "Bb" subset was selected (Available in preparative quantities, typically at least 250 mg) (2). Finally, the search was carried out clicking the "Search with" button and selecting the "Substructure" option (3).

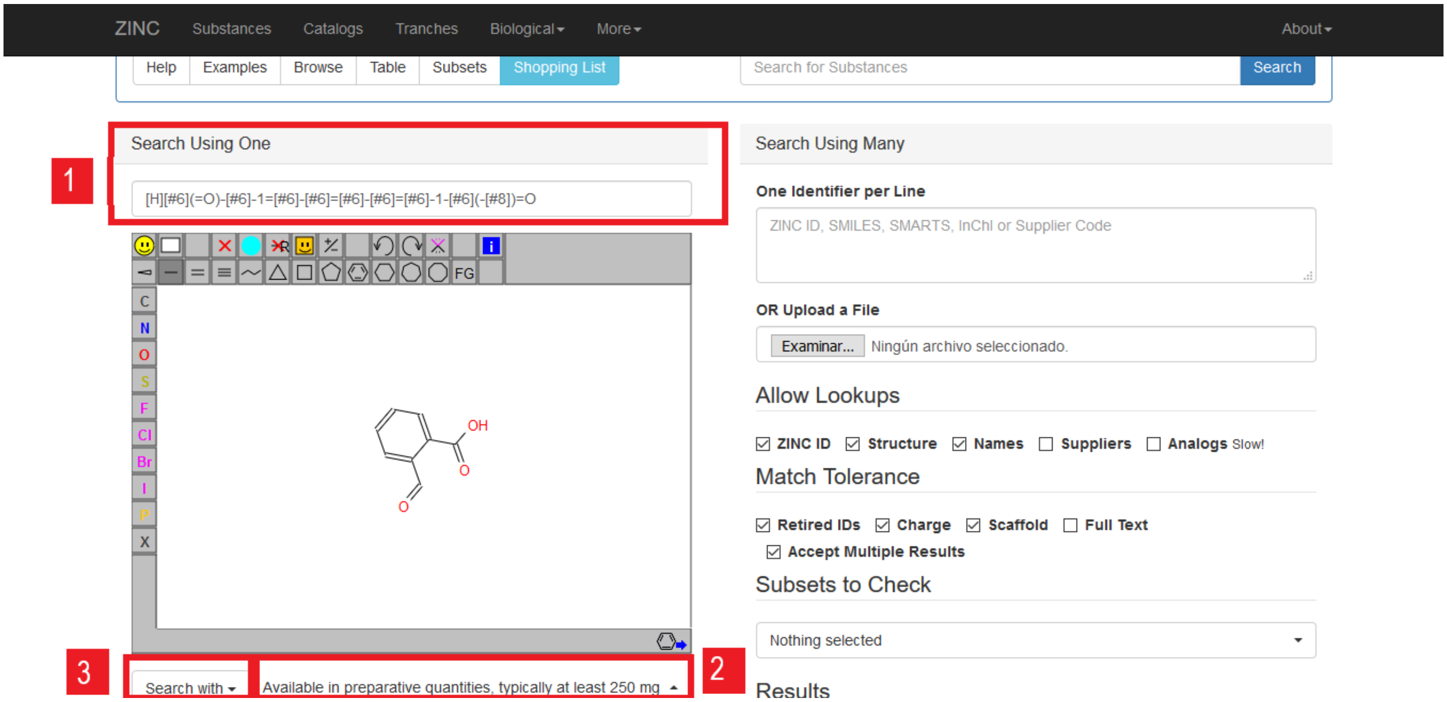

c) The database consisting of 72 compounds was downloaded on SDF format. Accessed: 12 Jul 2020.

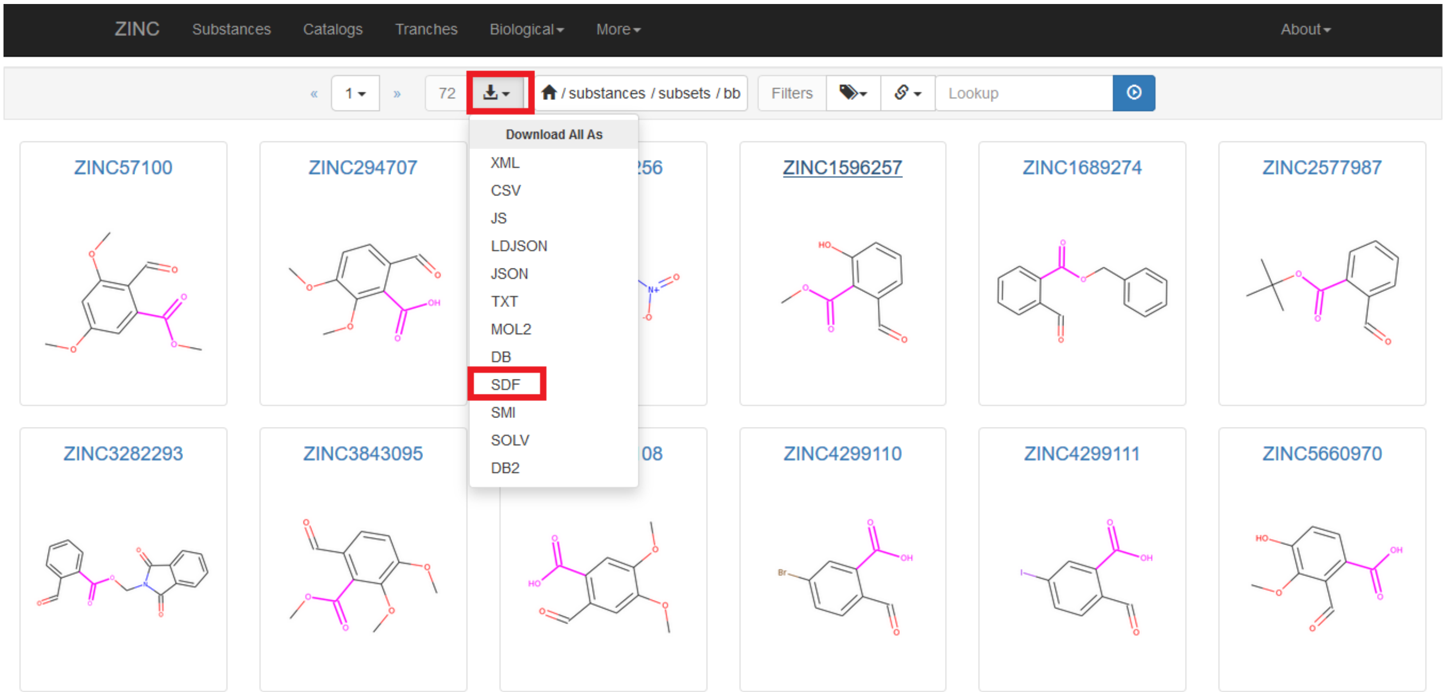

2. Filter parameters for Congreve's Rule of 3 used in FAF-Drugs server

Input parameters used in https://mobyle.rpbs.univ-paris-diderot.fr/cgi-bin/portal.py?form=FAF-Drugs4#forms::FAF-Drugs4 to filter the building blocks before them being used in Data Warrior.

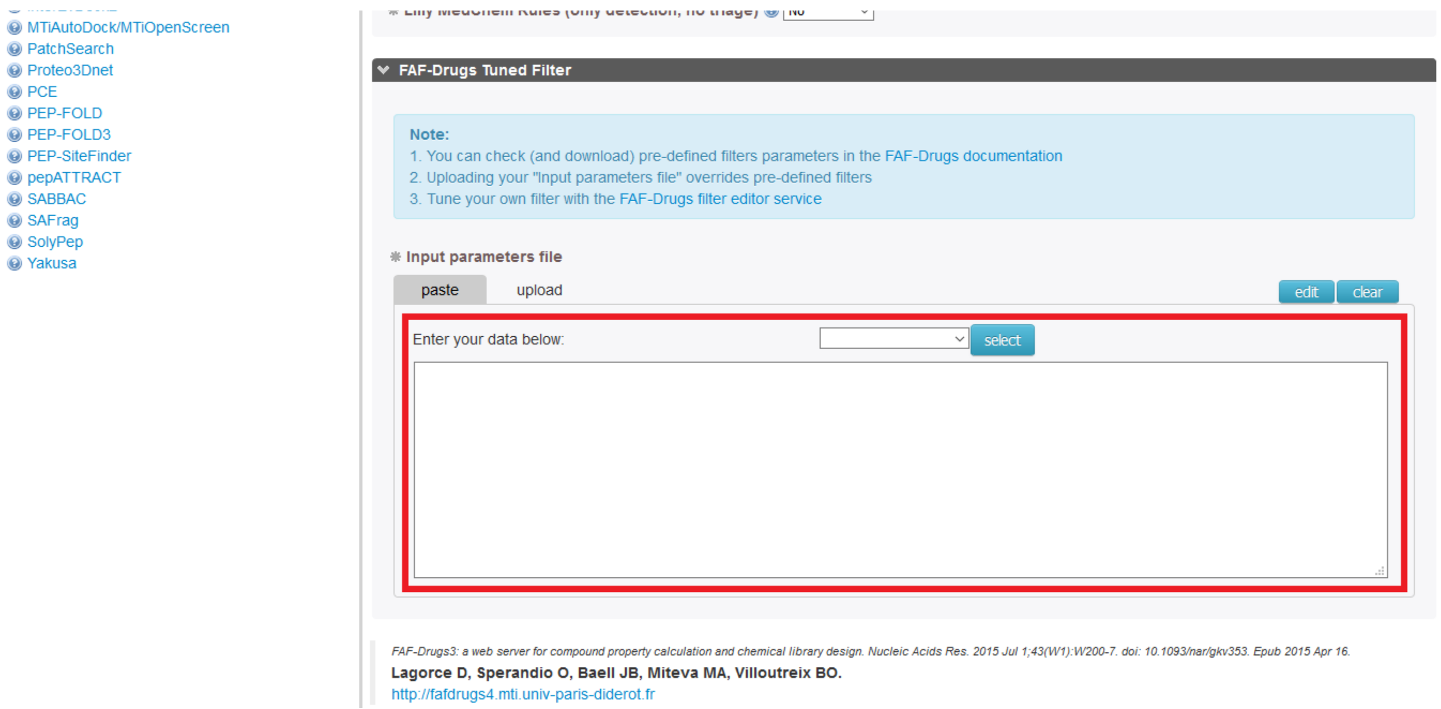


```
inf_mw = 0
inf_sc = None
inf_r = None
sup_logp = 3
sup_nc = None
inf_h = None
inf_nbb = 0
sup_hva = None
inf_c = None
sup_hbd = 3
inf_psa = 0
sup_hba = 3
inf_nrb = None
max_lipinski = None
max_ring = None
sup_nrb = None
inf_hva = None
sup_sc = None
sup_ch = None
inf_nc = None
inf_logp = 0
sup_mw = 300
sup_cf = None
sup_nbb = 3
sup_h = None
sup_psa = 60
sup_c = None
inf_ch = None
inf_hbd = 0
inf_hba = 0
inf_cf = None
sup_r = None
```

3. Substructure filtering in Data Warrior

a) Open the program DataWarrior (Available in: http://www.openmolecules.org/datawarrior/download.html)

b) Go to the menu File>Open to open the file "synquestecbb.sdf". Files accepted are: .CSV, .SDF, .TXT or .DWAR. Once the file is open, several windows will be displayed on your screen. The following figure describes the information and tools available in each of them.

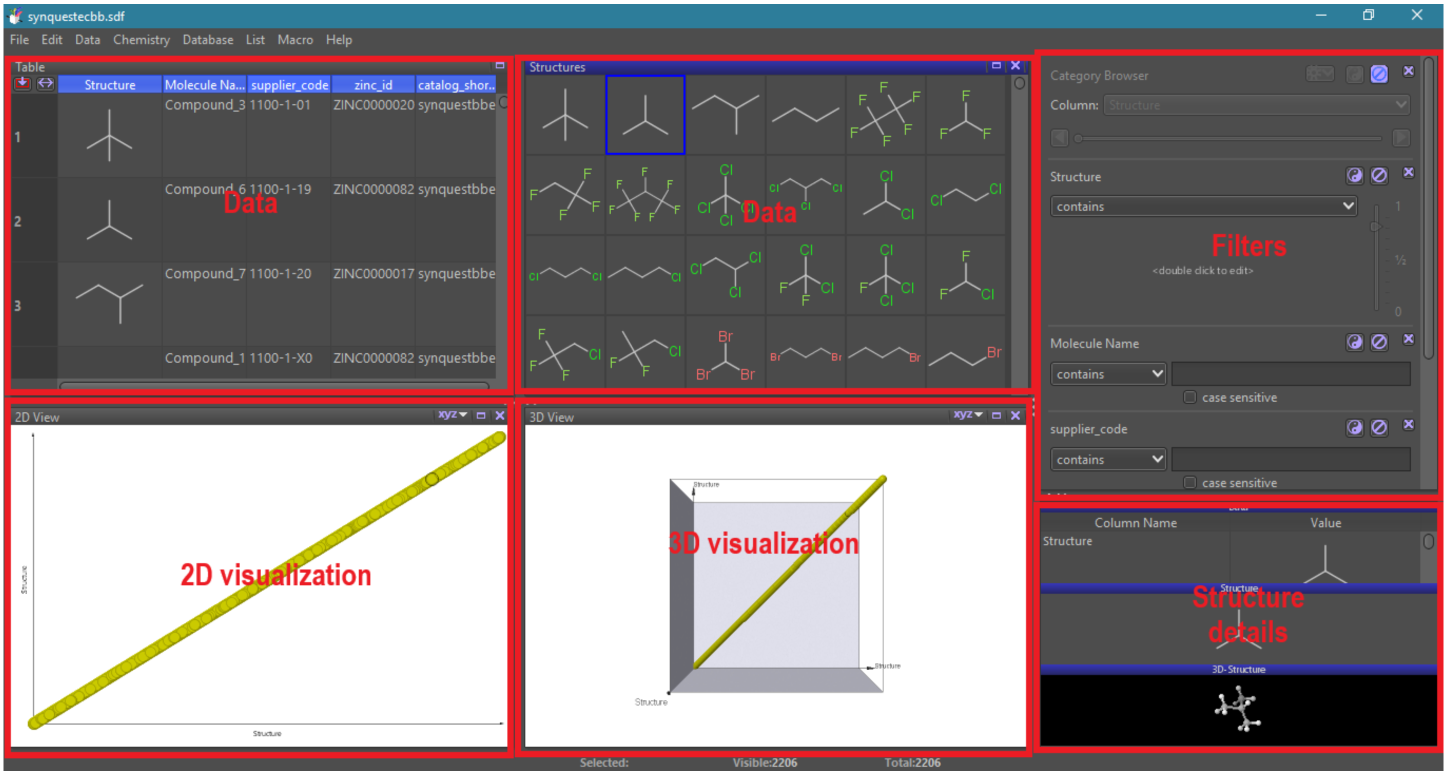

c) Right click on the "Structure" column to display options and go to New Structure Filter>Substructure List (1). A new filter option should appear on the filter area of the screen (2).

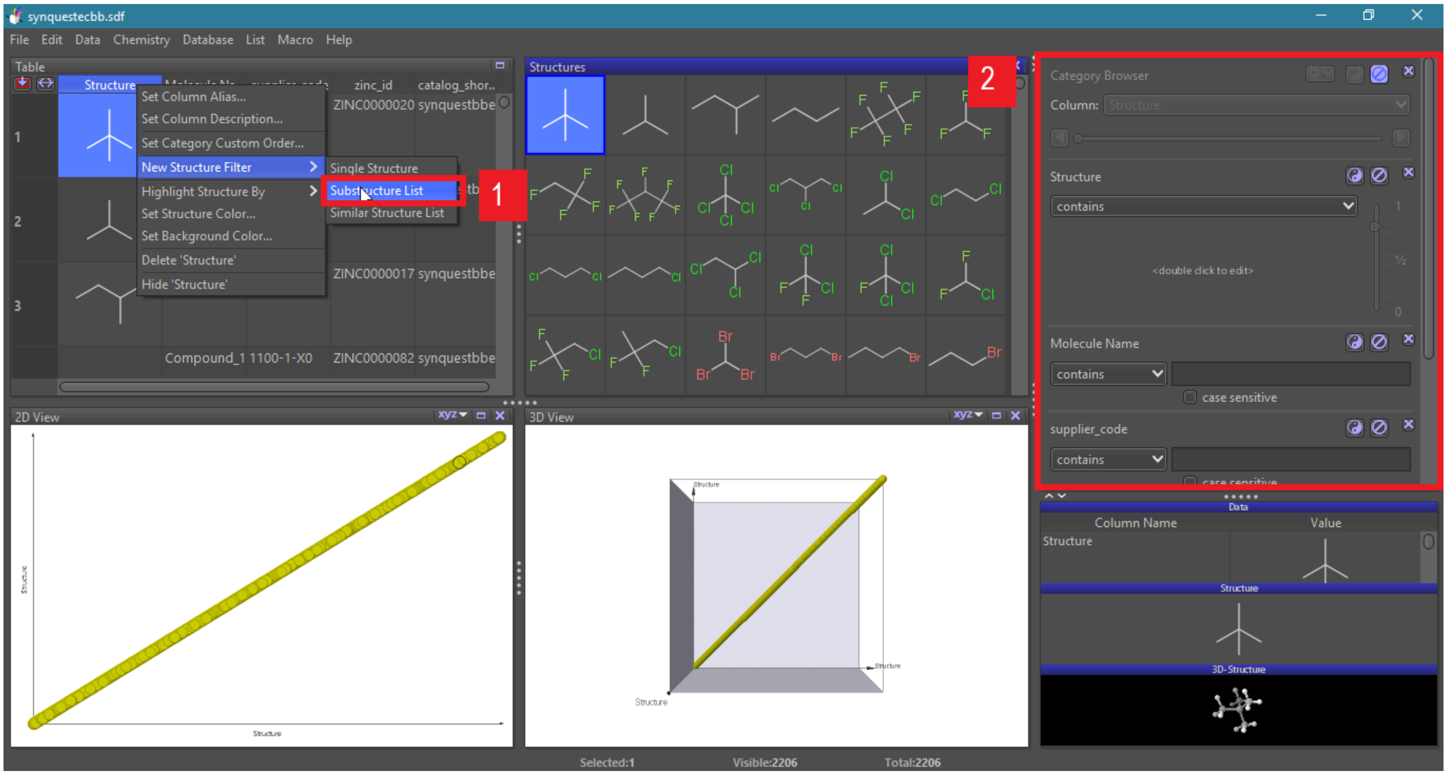

d) Right click on the empty area of the added filter to display options. Go to Add (1) Draw the desired functional group using the Structure Editor (2), the primary amine is shown as an example. A bond was drawn and a nitrogen was added as one of the atoms conforming the bond. Select the lasso function and double click on any of the atoms or bonds to assign desired properties (3). For primary amine, in the nitrogen atom hydrogen count was set to “exactly 2 hidrogens”, and for the carbon atom pi electron count was set to “no pi electrons”. Click OK button (4).

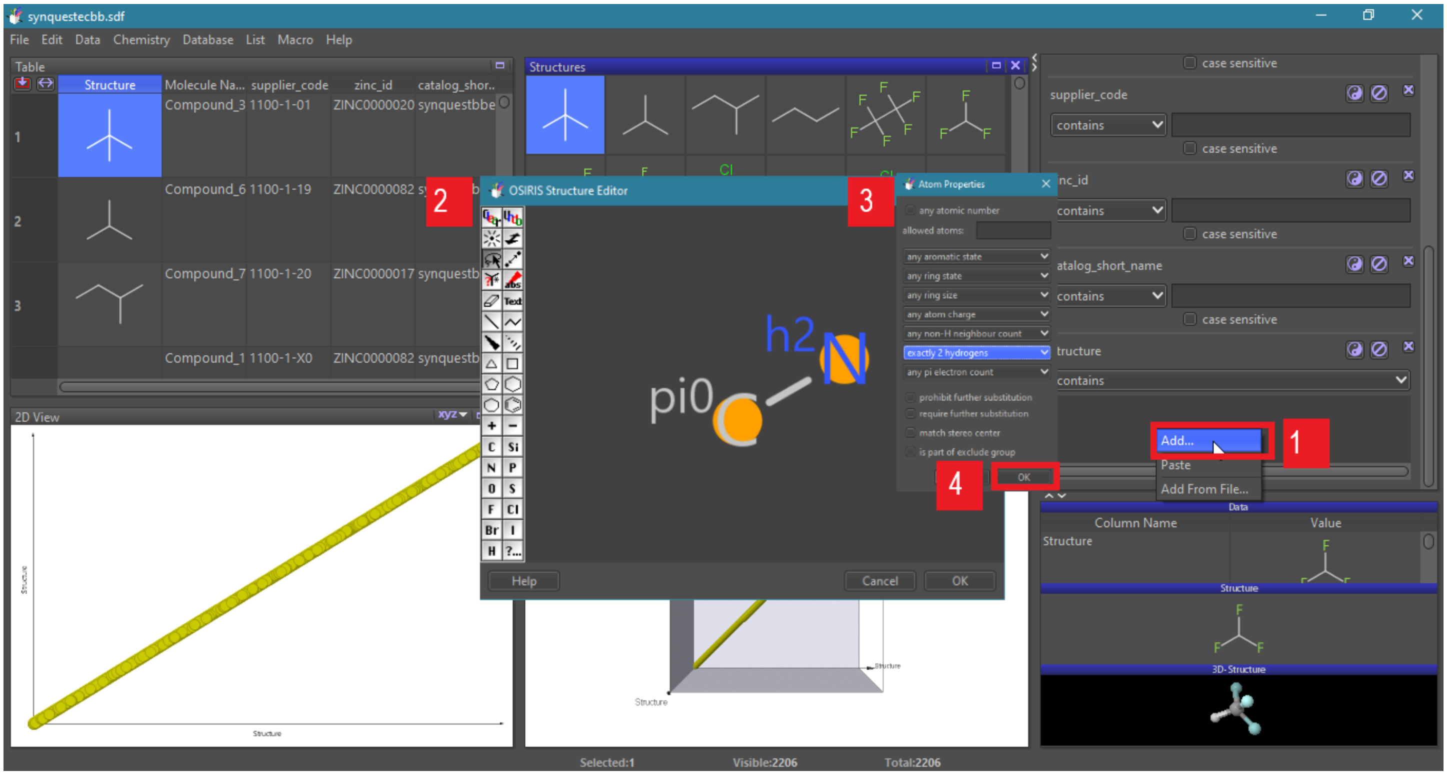

For the isocyanide the functional group was drawn assigning the correct charges to each atom (C^{-}, N^{+}). For the case of the 2-carboxybenzaldehyde, to ensure no further undesired substitution the carbon corresponding to the aldehyde was set to exactly one hydrogen and the oxygen of the carboxylic acid was also set to exactly one hydrogen, as displayed on the following figure.

Isocyanide filter	2-carboxybenzaldehyde filter
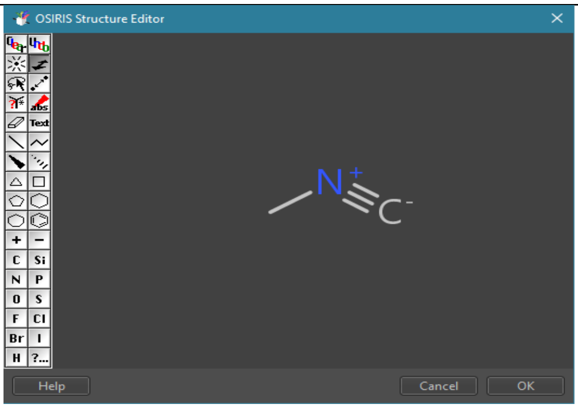	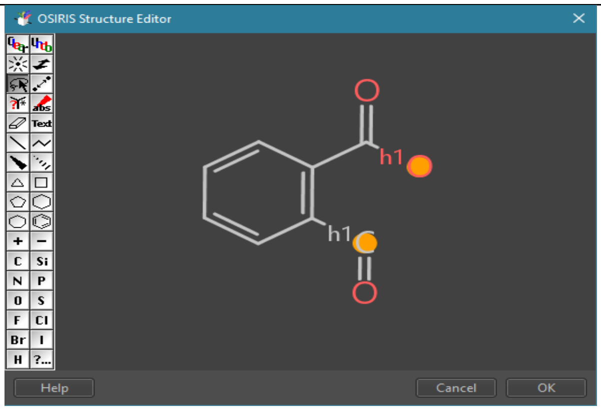

e) Go to Chemistry>Add Compound Properties (1). Go to Counts (2) and select “Aromatic Ring Count” (3). Click OK button (4).

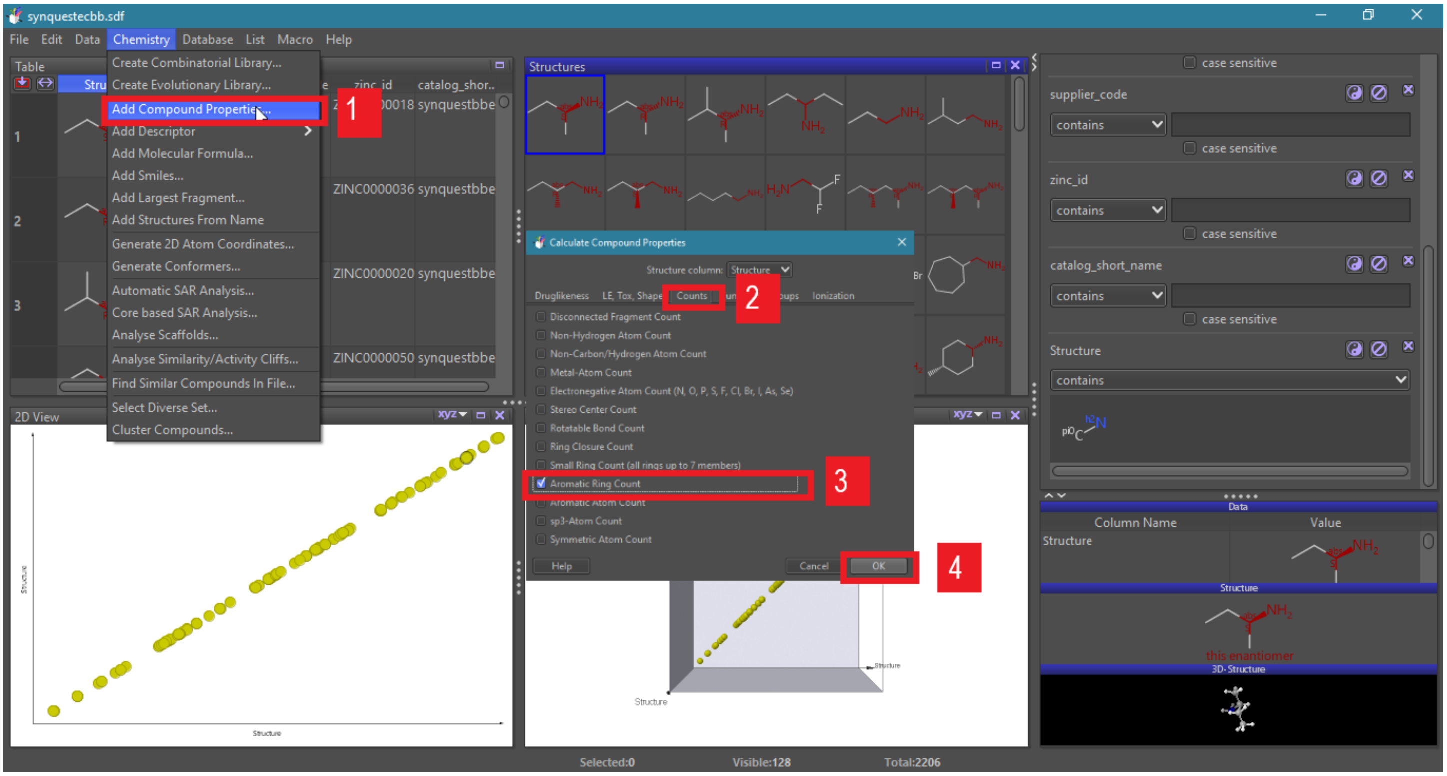

f) In the new Aromatic Rings filter added, set the value to 0.

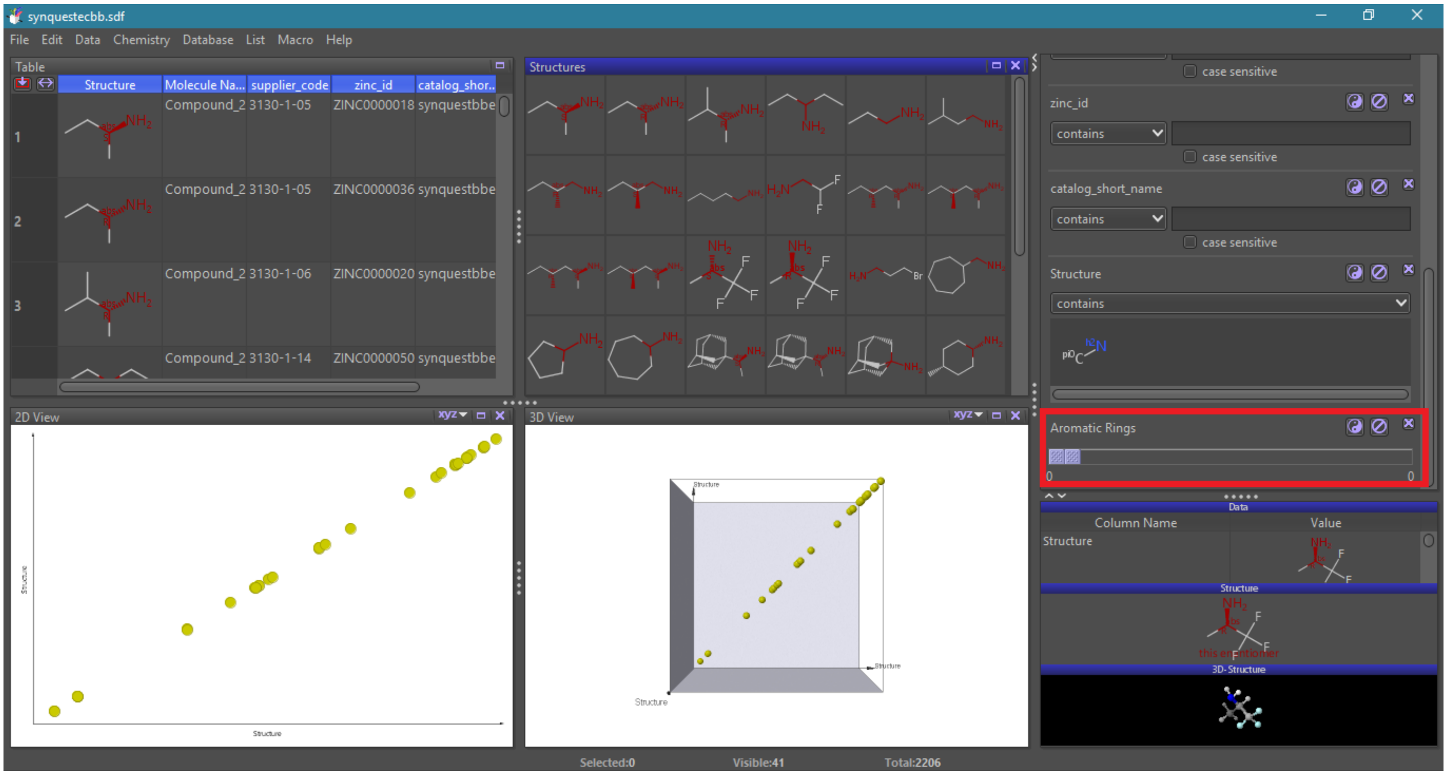

g) Select all the structures and go to File>New From Selection.

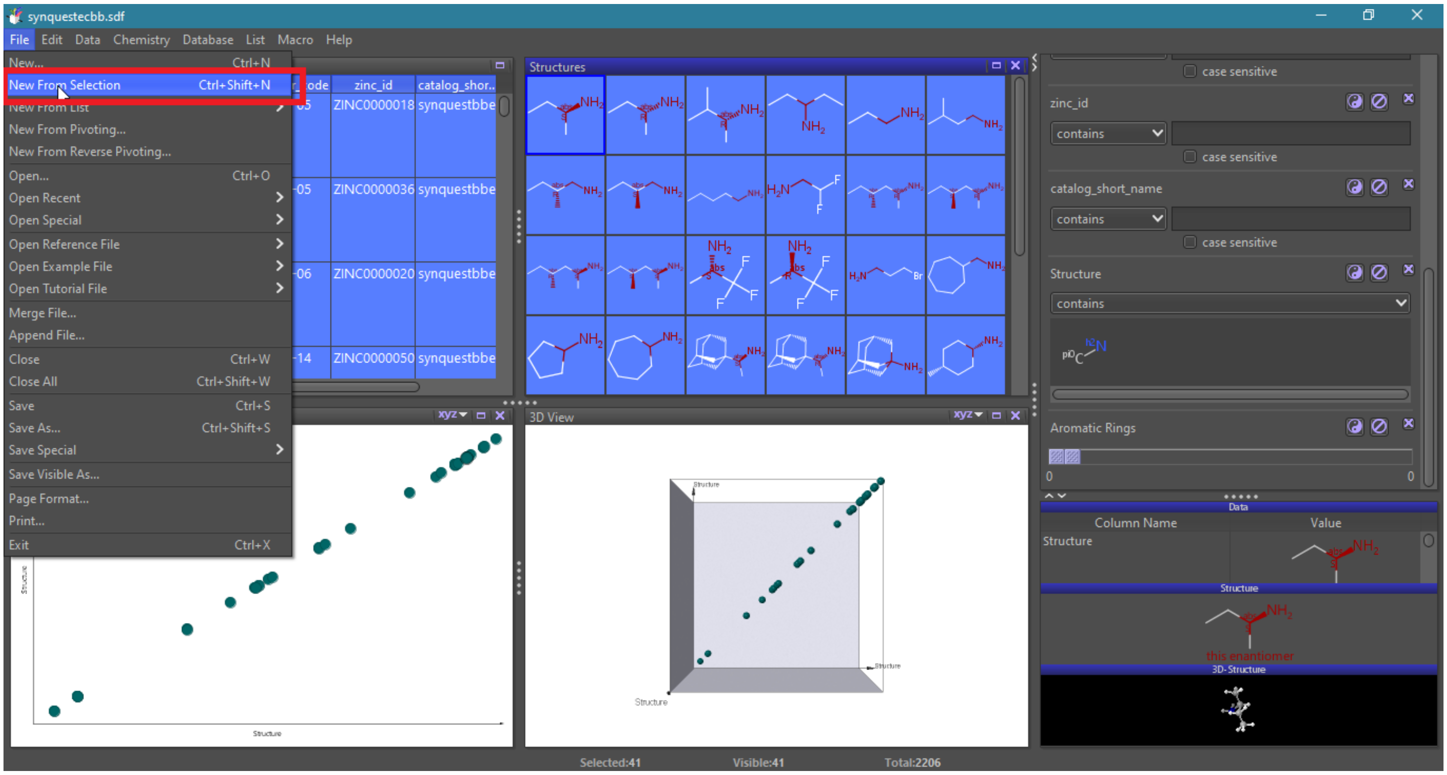

h) In the new file generated go to File>Save Special>SD-File.

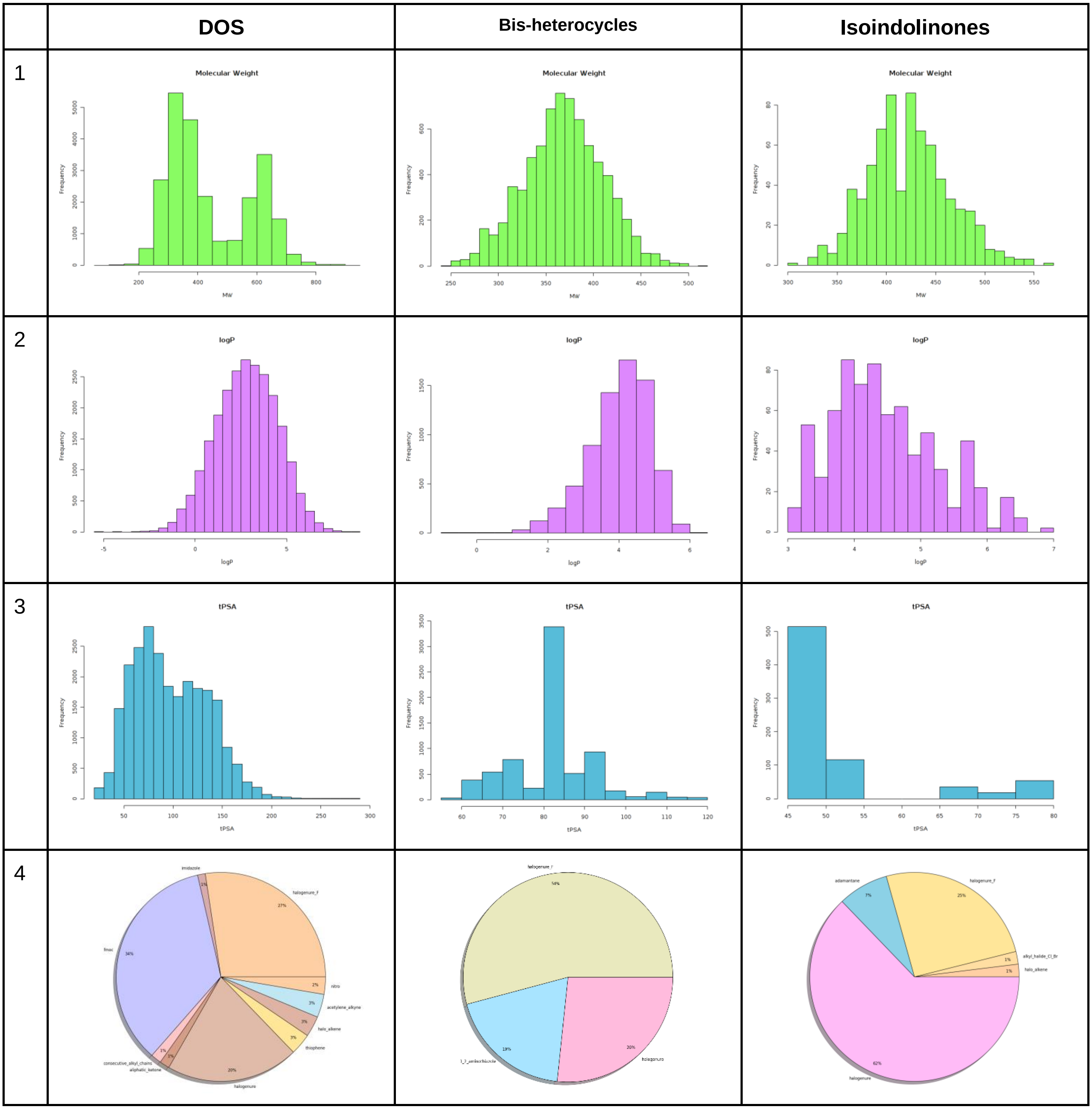

Figure S2. FAF-Drugs distribution of calculated properties and problematics moieties graphs from the built libraries. 1) Molecular weight. **2)** logP. **3)** TPSA. **4)** Problematic moieties (with an occurence above 1%).
